# Satellite DNA sequence dictates pericentromere heterochromatin formation and function

**DOI:** 10.1126/sciadv.ady2267

**Published:** 2026-07-15

**Authors:** Piero Lamelza, Malena Parrado, Kathleen Leara, Ahmed Z. Balboula, Thomas M. Keane, Michael A. Lampson

**Affiliations:** ^1^ Department of Biology, University of Pennsylvania, Philadelphia, PA, USA.; ^2^ Division of Animal Sciences, University of Missouri, Columbia, MO, USA.; ^3^ European Molecular Biology Laboratory, European Bioinformatics Institute, Wellcome Genome Campus, Hinxton, Cambridge, UK.; ^4^ Penn Center for Genome Integrity, University of Pennsylvania, Philadelphia, PA, USA.

## Abstract

Pericentromeres are heterochromatic regions adjacent to centromeres that ensure accurate chromosome segregation. Despite their conserved function, they are composed of rapidly evolving A/T-rich satellite DNA. To test the functional consequences of this rapid sequence evolution, we establish hybrid mouse embryos as a model system to compare divergent satellite arrays from distinct species in a common cytoplasm. We show that variation in satellite sequence impacts heterochromatin formation, recruitment of the Chromosome Passenger Complex (CPC), and interactions with the mitotic spindle. Differences in satellite DNA sequence alter pericentromere packaging by Polycomb Repressive Complex 1 (PRC1), as satellite arrays that recruit PRC1 are enriched for specific A/T sequences that the PRC1 AT-hook preferentially binds. Furthermore, PRC1 heterochromatin modifies pericentromere function by inhibiting recruitment of the CPC, increasing microtubule forces on kinetochores during mitosis. Our results provide a direct link between satellite DNA composition and mitotic chromosome behavior and highlight early embryogenesis as a critical point in development that is sensitive to satellite DNA evolution.

## Teaser

Rapidly evolving satellite DNA impacts heterochromatin formation and recruitment of a key regulator of chromosome segregation.

## INTRODUCTION

Satellite DNA is composed of tandemly repeated DNA sequences that make up large noncoding regions of eukaryotic genomes, e.g., 7 and 8% of human and mouse genomes, respectively (*1*, *2*). Satellites are among the most rapidly evolving genomic sequences, with their monomer size, nucleotide sequence, genomic distribution, and abundance varying widely between species (*3*–*6*). Recent advances in long-read sequencing technology have made huge progress in precisely characterizing the genetic composition and diversity of repetitive satellite arrays (*1*, *7*–*11*). However, it is still largely unknown what functional consequences may arise from the natural diversity of these sequences.

Despite their rapid evolution, satellites often underlie pericentromeres, heterochromatic loci adjacent to centromeres with conserved roles in chromosome segregation during cell division (*12*). This paradoxical observation is partially resolved by evidence indicating that pericentric heterochromatin maintenance and function are largely independent of satellite DNA sequence. Instead, factors that package and transcriptionally silence satellites rely on the recognition of histone 3 lysine 9 trimethylation (H3K9me3) marked nucleosomes that are canonically enriched at pericentromeres (*13*). Once established, H3K9me3 can propagate heterochromatin through successive cell cycles by recruiting histone methyltransferases that add new H3K9me3 (*13*). In addition, phosphorylation of pericentromeric histones during mitosis by Haspin and Bub1 kinases facilitates the recruitment of the chromosome passenger complex (CPC) (*14*), a key regulator of kinetochore-microtubule attachments required for accurate chromosome segregation (*15*, *16*). Because these epigenetic mechanisms do not directly depend on DNA sequence, potential functional impacts of variation in satellite DNA composition have been difficult to determine (*17*).

**Fig. 1.**
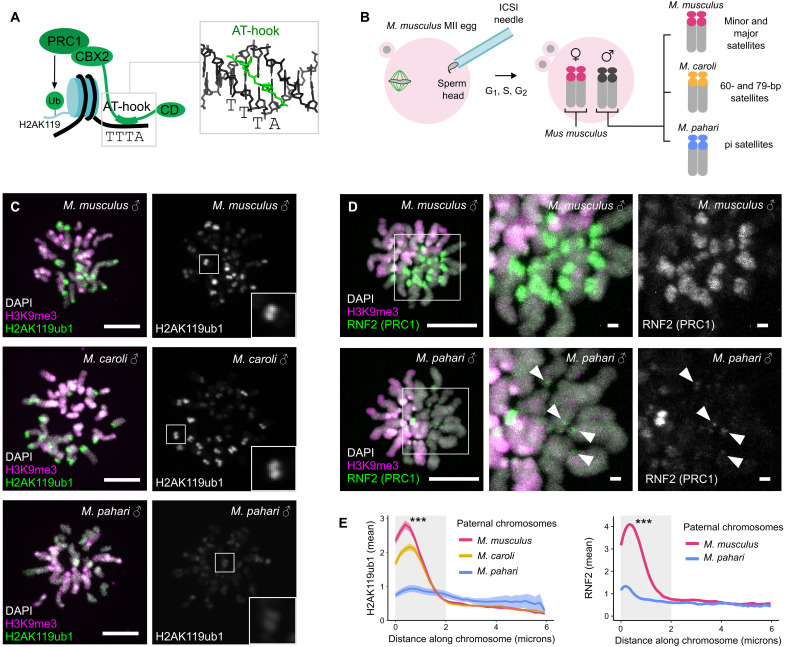
Divergence in pericentric H2AK119ub1 heterochromatin and PRC1 recruitment. (**A**) Schematic showing PRC1 recruitment to DNA via the AT-hook of its CBX2 subunit, which binds into the minor groove of A/T runs present in satellite DNA. Chromodomain labeled as “CD.” (**B**) Mouse zygotes are generated by injecting a *M. musculus* egg arrested in meiosis II with the sperm of mouse species with divergent pericentric satellite DNAs. Previously characterized satellites from each species are listed. (**C** to **E**) Zygotes generated with sperm from the indicated species were arrested in mitosis with a kinesin-5 inhibitor (STLC, C) or by APC/C inhibition (proTAME, D) and then fixed and stained for H2AK119ub1 or RNF2 (green), H3K9me3 (magenta) to mark maternal *M. musculus* chromosomes, and DAPI (gray). Arrowheads (D) highlight the slight enrichment of RNF2 near *M. pahari* centromeres. Graphs (E) show average H2AK119ub1 (*n* = 103 to 164, *N* = 2) and RNF2 (*n* = 152 to 160, *N* = 2) intensities along *M. musculus* (red), *M. caroli* (yellow), and *M. pahari* (blue) paternal chromosomes, starting from pericentric ends. SEM is indicated by light band surrounding the mean line. Both *M. musculus* and *M. caroli* chromosomes have significantly higher H2AK119ub1 intensity than *M. pahari* chromosomes within the first two microns (gray box) (***P < 0.001). Statistical significance was calculated by a Kruskal-Wallis test, followed by a Dunn’s post hoc test with Bonferroni correction. Images are max intensity z-projections. Scale bars, 10 or 1 μm (insets).

Mouse embryos provide an attractive model system to test the consequences of satellite DNA evolution on pericentromeric heterochromatin formation and function. Because most histones are replaced by protamines in mouse sperm, the paternal genome must undergo a drastic repackaging into nucleosome-based chromatin after fertilization (*18*, *19*). These nucleosomes lack H3K9me3, creating a unique developmental window in which canonical epigenetic pathways of heterochromatin maintenance are absent on paternal satellites (*20*). Instead, Polycomb Repressive Complexes 1 and 2 (PRC1 and PRC2) package paternal satellites in heterochromatin marked with H2AK119ub1 and H3K27me3, respectively, which typically suppress gene expression elsewhere in the genome (*20*–*22*). Both modifications contribute to the transcriptional silencing of the 240-bp A/T-rich “major satellite” repeats at *Mus musculus* pericentromeres (*20*, *23*), with PRC1 also crucial for maintaining the integrity of these arrays (*24*). Furthermore, both complexes can localize to pericentromeres by recognition of satellite DNA (*21*, *22*). The CBX2 subunit of PRC1 recognizes satellites via its AT-hook, a five–amino acid motif with nanomolar binding affinity for the relatively narrow minor grooves of double-stranded DNA (dsDNA) generated by stretches of tandem A/T nucleotides (A/T runs) (Fig. 1A) (*25*–*29*). PRC2 localization to paternal pericentromeres likely requires BEN domain containing 3 (BEND3), a methylation sensitive DNA binding protein that can bind unmethylated major satellite DNA in vitro (*21*).

We developed a system to test whether variation in satellite DNA sequence alters paternal heterochromatin by fertilizing *M. musculus* eggs with sperm from closely related mouse species with divergent satellite repeats. We inject sperm into egg cells [intracytoplasmic sperm injection (ICSI) (*30*)] to bypass fertilization barriers between species, allowing us to test satellite DNA variation that would otherwise be inaccessible by natural mating. We focus our analysis on the one-cell zygote, before the major transcriptional activation of the mouse embryo (*31*), so that our results reflect interactions between satellite sequences and maternally inherited *M. musculus* Polycomb complexes rather than a mixture of subunits from both species. Analyzing heterochromatin in the zygote also avoids the complication of potential genetic incompatibilities that typically arise after the transcriptional activation of the hybrid genome (*32*–*34*).

We demonstrate that variation in satellite DNA sequence significantly influences PRC1 recruitment and heterochromatin formation at pericentromeres. Satellite arrays that robustly recruit PRC1 are enriched for specific A/T runs (i.e., AAAAT) that optimally bind the CBX2 AT-hook based on previous reports of AT-hook proteins (*29*) and our AlphaFold3 modeling. We find that PRC1 heterochromatin modifies pericentromere function by inhibiting the recruitment of the CPC to pericentromeres. The CPC ensures accurate chromosome segregation by regulating chromosome attachment to the mitotic spindle by destabilizing kinetochore-microtubule interactions. Pericentromeres that form PRC1 heterochromatin exhibit longer sister kinetochore distances during metaphase, consistent with a reduction in CPC stabilizing kinetochore-microtubule interactions and increasing the force pulling sister kinetochores apart. Furthermore, we find that paternal chromosomes with PRC1 heterochromatin are more prone to missegregate during anaphase compared to maternal chromosomes, which lack PRC1. Our results show that diverse pericentric DNA sequences lead to functionally diverse pericentromeres through differential binding of heterochromatin forming complexes.

## RESULTS

### Variation in PRC1 localization and heterochromatin formation on divergent satellites

Given that major satellite DNA can direct PRC1 and PRC2 heterochromatin formation, we hypothesized that divergent pericentric satellite sequences might alter CBX2 or BEND3 binding and by extension H2AK119ub1 or H3K27me3 formation, respectively. We fertilized *M. musculus* eggs with sperm from mouse species harboring genetically divergent pericentric satellites (Fig. 1B). We selected three species (*M. musculus*, *Mus caroli*, and *Mus pahari*) that differ in their satellite monomer sequence and not only in satellite copy number as seen in *M. musculus* subspecies (*2*, *5*). We injected sperm heads into MII eggs, arrested the resulting zygotes at the first mitosis by kinesin-5 inhibition, and measured H2AK119ub1 and H3K27me3 by immunofluorescence. Because only maternal chromosomes have H3K9me3 at this stage, we used this mark to distinguish maternal and paternal chromosomes (*20*).

We found that all three species’ pericentromeres robustly acquire H3K27me3 (fig. S1, A and B). In contrast, H2AK119ub1 is enriched on paternal *M. musculus* and *M. caroli* pericentromeres relative to chromosome arms but not on *M. pahari* pericentromeres (Fig. 1, C and E). To test whether these differences result from differences in PRC1 localization, we measured PRC1’s core-catalytic subunit, Ring finger protein 2 (RNF2), by immunofluorescence. Consistent with the H2AK119ub1 enrichment, paternal *M. musculus* pericentromeres are strongly enriched for RNF2 relative to chromosome arms, whereas *M. pahari* chromosomes only exhibit a slight enrichment near the very ends of their chromosomes, presumably near centromeres (Fig. 1, D and E).

In certain contexts, PRC1 is recruited to chromatin by the chromodomain of its CBX subunit, which directly binds H3K27me3 (*35*–*37*), and CBX2 also contains a chromodomain (Fig. 1A). However, the presence of H3K27me3 at *M. pahari* paternal pericentromeres (fig. S1, A and B), which lack H2AK119ub1 and RNF2 (Fig. 1, C to E), indicates that H3K27me3 is not sufficient for PRC1 recruitment during the first zygotic mitosis. In contrast to mitosis, both H3K27me3 and RNF2 are enriched at both *M. musculus* and *M. pahari* paternal pericentromeres during G_2_ (fig. S1, C to E), consistent with previous reports that the CBX2 chromodomain contributes to PRC1’s pericentric localization during zygotic interphase (*22*). The difference in RNF2 localization between G_2_ and mitosis indicates that the CBX2 chromodomain does not effectively localize PRC1 during mitosis. We speculate that Aurora B–mediated phosphorylation of H3S28 during mitosis (*38*) prevents the CBX2 chromodomain from binding H3K27me3, similar to how Aurora B–mediated phosphorylation of H3S10 inhibits HP1 chromodomain binding to H3K9me3 (*39*, *40*). Therefore, the observed differences between *M. musculus* and *M. pahari* paternal pericentromeres during mitosis, in both PRC1 recruitment and heterochromatin formation, likely reflect variation among the divergent satellite DNA sequences in their ability to bind the CBX2 AT-hook.

### Pericentromeres enriched for narrow DNA minor grooves form PRC1 heterochromatin

**Fig. 2.**
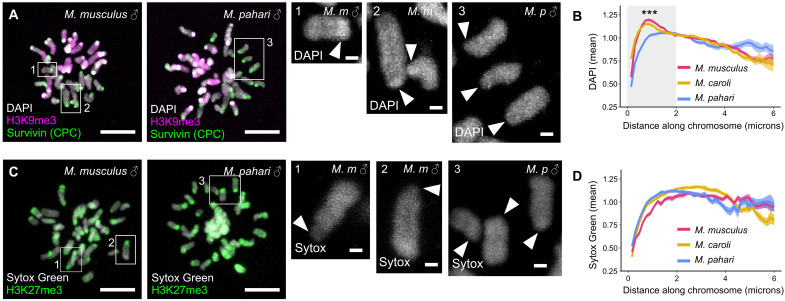
Paternal pericentromeres that form PRC1 heterochromatin are enriched for narrow DNA minor grooves. (**A** to **D**) Zygotes generated with *M. musculus* or *M. pahari* sperm were arrested in mitosis with a kinesin-5 inhibitor (STLC) and then fixed and stained as indicated. Survivin (CPC subunit) marks pericentromeres, H3K9me3 marks maternal chromosomes, H3K27me3 marks paternal pericentromeres, and DAPI or Sytox Green labels DNA. Arrowheads in insets point to paternal pericentromeres as indicated by the presence of Survivin and the absence of H3K9me3 (A) or enrichment of H3K27me3 (C). Images are max intensity z-projections. Scale bars, 10 or 1 μm (insets). Graphs show average DAPI (B, *n* = 72 to 165, *N* = 2) or Sytox Green (D, *n* = 44 to 66, *N* = 2) intensity along *M. musculus* (red), *M. caroli* (yellow), and *M. pahari* (blue) paternal chromosomes, starting from pericentric ends. Both *M. musculus* and *M. caroli* chromosomes have significantly higher DAPI intensity than *M. pahari* chromosomes within the first two microns (B, gray box) (***P < 0.001). Statistical significance was calculated by a Kruskal-Wallis test, followed by a Dunn’s post hoc test with Bonferroni correction.

To explain the differences in pericentric RNF2 and H2AK119ub1, we hypothesized that *M. musculus* and *M. caroli* pericentromeric satellites, but not *M. pahari* satellites, are enriched for narrow DNA minor grooves that bind the AT-hook of CBX2 to recruit the PRC1 complex. Following a previously described approach (*2*), we used 4′,6-diamidino-2-phenylindole (DAPI) staining to compare the amount of narrow minor grooves generated by A/T runs at each species’ pericentromere, as DAPI specifically binds to these regions on the dsDNA molecule (*41*–*43*). Consistent with previous observations (*2*, *9*, *44*), we find increased DAPI staining at *M. musculus* and *M. caroli* pericentromeres relative to chromosome arms, but not at *M. pahari* pericentromeres (Fig. 2, A and B, and fig. S2A). To exclude the possibility that DAPI enrichment at pericentromeres represents a relatively more compact chromatin state compared to the rest of the chromosome, we stained with Sytox Green, an intercalating DNA dye with no considerable DNA sequence preference (*45*). None of the three species show enrichment of Sytox Green at their pericentromeres (Fig. 2, C and D, and fig. S2B). Together, these results demonstrate that *M. musculus* and *M. caroli* pericentromeres are enriched for narrow A/T-rich minor grooves that can robustly bind the AT-hook of CBX2, whereas *M. pahari* pericentromeres are not.

### Satellite sequence determinants of PRC1 binding

**Fig. 3.**
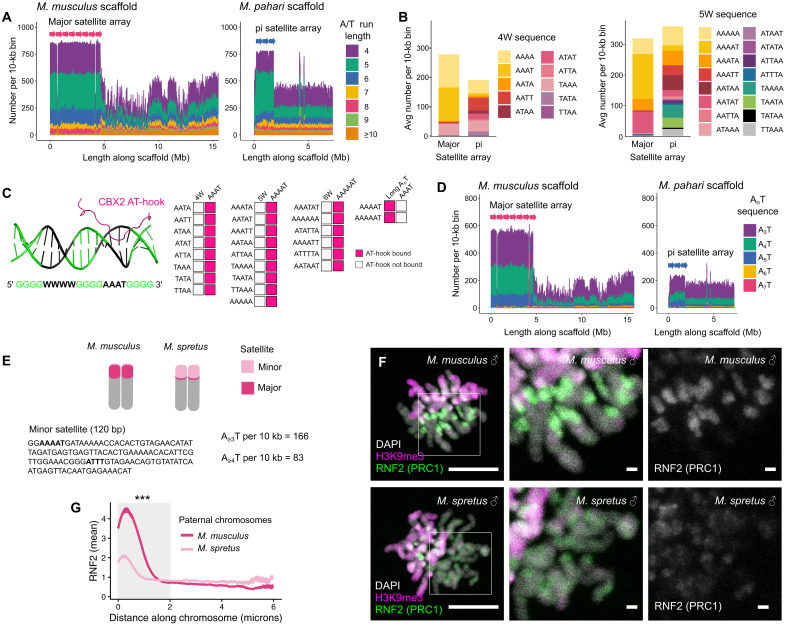
*M. musculus* major satellite arrays are enriched for long A_n_T sequences that preferentially bind the CBX2 AT-hook. (**A**) Histograms show the number of various A/T run lengths per 10-kb bin along portions of two genomic scaffolds containing arrays of either *M. musculus* major satellite (left) or *M. pahari* pi satellite (right), indicated by tandem arrows. (**B**) The average number per 10-kb bin of all possible 4W (left) and 5W (right) sequences within the major and pi satellites arrays shown in (A). (**C**) Schematic (left) shows in silico competition assays between different A/T run sequences for the binding of a single peptide encoding the AT-hook of CBX2, using AlphaFold3. Results (right) show AT-hook binding preferences. (**D**) Histograms show the number of various A_n_T sequences per 10-kb bin along the same genomic scaffolds as in (A). (**E**) Top: schematic showing that *M. musculus* and *M. spretus* centromeres/pericentromeres have inverse proportions of minor (pink) and major (red) satellites. Bottom: consensus minor satellite sequence with A_n_T sequences in bold and predicted A_n_T frequencies. (**F**) Zygotes generated with *M. musculus* (top row) or *M. spretus* (bottom row) sperm were arrested in mitosis by APC/C inhibition (proTAME) and then fixed and stained for RNF2 (green), H3K9me3 (magenta) to mark maternal *M. musculus* chromosomes, and DAPI (gray). Images are max intensity z-projections. Scale bars, 10 or 1 μm (insets). (**G**) Graphs show average RNF2 intensity along *M. musculus* (red, *n* = 166) and *M. spretus* (pink, *n* = 119) paternal chromosomes, starting from pericentric ends. SEM is indicated by light band surrounding the mean line*. M. musculus* chromosomes have significantly higher RNF2 intensity than *M. spretus* chromosomes within the first two microns (gray box) (***P < 0.001) (*N* = 2). Statistical significance was calculated by a Kruskal-Wallis test, followed by a Dunn’s post hoc test with Bonferroni correction.

We considered two possibilities for the underlying differences in pericentric DNA sequence between species, leading to differences in DAPI enrichment and PRC1 localization. We initially focused on comparisons between *M. musculus* and *M. pahari* because of the long-read sequencing data available for these species. First, *M. musculus* major satellites may have a higher frequency of A/T runs compared to *M. pahari* pericentric satellites (pi satellites) (*9*). However, major and pi satellite consensus sequences have similar frequencies of A/T runs of lengths ranging from 4 (4W, the minimal number of nucleotides the AT-hook can span) to 10 or more (≥10W) (fig. S2C). To capture nucleotide diversity that may be lost in consensus sequences, we also calculated the frequencies of A/T runs across genomic scaffolds containing either major or pi satellite arrays. Consistent with consensus sequences, we find that both species’ satellite arrays have similar frequencies of A/T runs (~825 versus ~750 per 10 kb bin in major satellite or pi satellite, respectively) (Fig. 3A). The frequencies are also similar for each length of A/T runs (Fig. 3A and fig. S2D), with the exceptions of 6W and ≥10W, which are relatively more abundant in major and pi satellite arrays, respectively. However, it is unclear how differences in these lengths specifically would lead to higher PRC1 and DAPI at *M. musculus* major satellite arrays. Therefore, it is unlikely that differences in A/T run frequency explain the difference in PRC1 heterochromatin formation.

The second possibility is that *M. musculus* major satellite arrays might be enriched for specific A/T sequences optimal for CBX2 AT-hook binding, compared to *M. pahari* pi satellite arrays. We analyzed the sequence composition of 4W, 5W, and 6W A/T runs, which make up ~75% of the A/T stretches in both species’ satellite arrays and therefore most potential AT-hook binding sites. The vast majority of 4W sequences in *M. musculus* major satellite arrays are AAAT (114 per 10 kb), AAAA (113 per 10 kb), or TAAA (39 per 10 kb) (Fig. 3B and fig. S2E). *M. pahari* pi satellite arrays have a similar frequency of TAAA sites (37 per 10 kb) but much lower frequencies of AAAT and AAAA sites (7.2 and 46 per 10 kb, respectively). One of these sequences, AAAT, generates one of the narrowest minor grooves compared to other tetranucleotides (*46*), suggesting preferential binding to an AT-hook. In addition, the core motif of the AT-hook (PRGRP) preferentially binds the minor groove of the sequence 5′ AA(A/T)T 3′, reflecting optimal van der Waals packing when adenine bases are on opposite sides of the A/T run (*29*). Because of these structural features and their relative abundance in major satellite, we hypothesized that the CBX2 AT-hook may preferentially bind the narrow minor groove of AAAT.

To test the binding preferences of the CBX2 AT-hook, we used AlphaFold3 (*47*) to perform in silico pairwise competitive binding assays for AAAT versus all other possible A/T tetranucleotides (Fig. 3C). We first confirmed that AlphaFold3 accurately models AT-hook binding to dsDNA using the well-characterized HMGA1 AT-hook bound to a fragment of the interferon-β promoter (*28*) (Protein Data Bank ID 2EZD) as a reference. The predicted structure closely matched the known structure (root mean square deviation = 0.48 Å), and in both structures, the AT-hook occupied the minor groove of the A/T run (AAATT) (fig. S2F). Furthermore, the confidence scores of the predicted structure [Interface Predicted Template Modeling Score (iPTM) = 0.48 and Predicted Template Modeling Score (PTM) = 0.47] are similar to the models generated in our in silico competitive binding assay (table S1). The AT-hooks of CBX2 and HMGA1 share the same core sequence (PRGRP) which binds the minor groove, supporting our use of AlphaFold3 to model the AT-hook binding preference of CBX2.

For each competition assay, we model a single dsDNA molecule encoding two distinct A/T runs flanked by four G:C base pairs (5′GGGGWWWWGGGGWWWWGGGG 3′). These two A/T runs “compete” to bind a single peptide consisting of the core CBX2 AT-hook along with flanking amino acids (RKRGKR**PRGRP**RKHTVTSS, AT-hook in bold). All AlphaFold models predict that the CBX2 AT-hook prefers to bind AAAT over almost all other A/T tetranucleotides (Fig. 3C). When AAAT and AAAA compete against each other, their relative order on the dsDNA dictates which sequence binds the AT-hook (fig. S2G; see Materials and Methods), indicating that these two sequences have similar affinity for the AT-hook. We obtain similar results when analyzing 5W and 6W A/T runs. AAAAT and AAAAAT are the most abundant 5W and 6W sequences in the *M. musculus* major satellite array (147 per 10 kb and 70 per 10 kb, respectively) (Fig. 3B and fig. S2E). These sequences outcompete all other prevalent 5W and 6W sequences in both major and pi satellite arrays for CBX2 AT-hook binding (Fig. 3C and fig. S2G). Furthermore, AAAAT and AAAAAT are 8- and 2.5-fold less frequent, respectively, in *M. pahari* pi satellite compared to *M. musculus* major satellite arrays (Fig. 3B and fig. S2E). We also find that AAAAT has one of the narrowest minor grooves of the analyzed A/T runs (fig. S3), consistent with this feature enhancing AT-hook binding (*29*), indicating that the AlphaFold3 models capture empirical principles governing AT-hook–DNA interactions.

Our AlphaFold3 modeling shows that the CBX2 AT-hook prefers to bind stretches of adenine nucleotides that end with a single thymine (A_n_T). To extend this analysis to include A_n_T sequences (like AAAT) present in longer A/T runs, we quantified the frequency of A_n_T sequences of various lengths (i.e., A_3_T, A_4_T, A_5_T, A_6_T, and A_7_T) in major and pi satellite arrays independent of total A/T run length. We find that major satellite arrays have approximately three times more A_n_T sequences than *M. pahari* pi satellite arrays (Fig. 3D and fig. S2H). Furthermore, pi satellite arrays exhibit A_n_T frequencies similar to chromosome arms. Within major satellite arrays, longer A_n_T sequences (i.e., A_4_T and A_5_T) are more enriched relative to chromosome arms (roughly 5.3-fold and 6.8-fold, respectively) than A_3_T (roughly 2.5-fold) (Fig. 3D, fig. S2H, and table S2). Our in silico analysis shows that A_4_T and A_5_T outcompete A_3_T for CBX2 AT-hook binding (Fig. 3C), consistent with previous findings that longer A/T runs are preferred binding sites for AT-hook proteins (*48*). Overall, these analyses indicate that PRC1 and its H2AK119ub1 modification are enriched at *M. musculus* major satellite arrays because they are enriched for longer A_n_T sequences that preferentially bind the CBX2 AT-hook. In contrast, *M. pahari* pi satellite arrays have fewer and shorter A_n_T sequences, like chromosome arms, and fail to enrich both PRC1 and H2AK119ub1.

To further test our model, we compared PRC1 recruitment between *M. musculus* and *Mus spretus* paternal pericentromeres. Both species harbor minor and major satellite arrays but in roughly inverse proportions. Major and minor satellites make up roughly ~7.9 and ~0.5% of the *M. musculus* genome, respectively, compared to ~0.2 and ~4.4% in *M. spretus* (Fig. 3E) (*2*). Although long-read assemblies of *M. spretus* are not currently available, the minor satellite consensus sequence is not enriched for A_n_T sequences (Fig. 3E). We therefore predicted that the abundant minor satellites at *M. spretus* pericentromeres would not recruit PRC1 as strongly as the major satellites enriched *M. musculus* pericentromeres. We find that paternal *M. musculus* pericentromeres recruit roughly twofold more RNF2 than paternal *M. spretus* pericentromeres (Fig. 3, F and G). This result demonstrates that the A_n_T-enriched major satellite is the primary driver of PRC1 recruitment to *M. musculus* pericentromeres, based on direct comparison of species whose pericentromeres primarily differ in the relative abundance of the same two satellite sequences.

### AlphaFold modeling predicts a previously unidentified A_n_T-rich *M. caroli* satellite

Because *M. caroli* pericentromeres form PRC1 heterochromatin (Fig. 1C), our model predicts that *M. caroli* satellites should be enriched for A_n_T sequences. However, analysis of the known *M. caroli* satellites (*49*) shows that the 60-bp satellite lacks A_n_T sequences, while the 79-bp satellite contains only A_3_T at a low frequency comparable to *M. pahari* pi satellite (~200 per 10 kb) (fig. S4A). Thus, these *M. caroli* satellites are not enriched for the longer A_n_T sequences found in major satellite. We therefore hypothesized that *M. caroli* has additional, previously uncharacterized satellites containing longer A_n_T sequences that are responsible for PRC1 recruitment and heterochromatin formation.

**Fig. 4.**
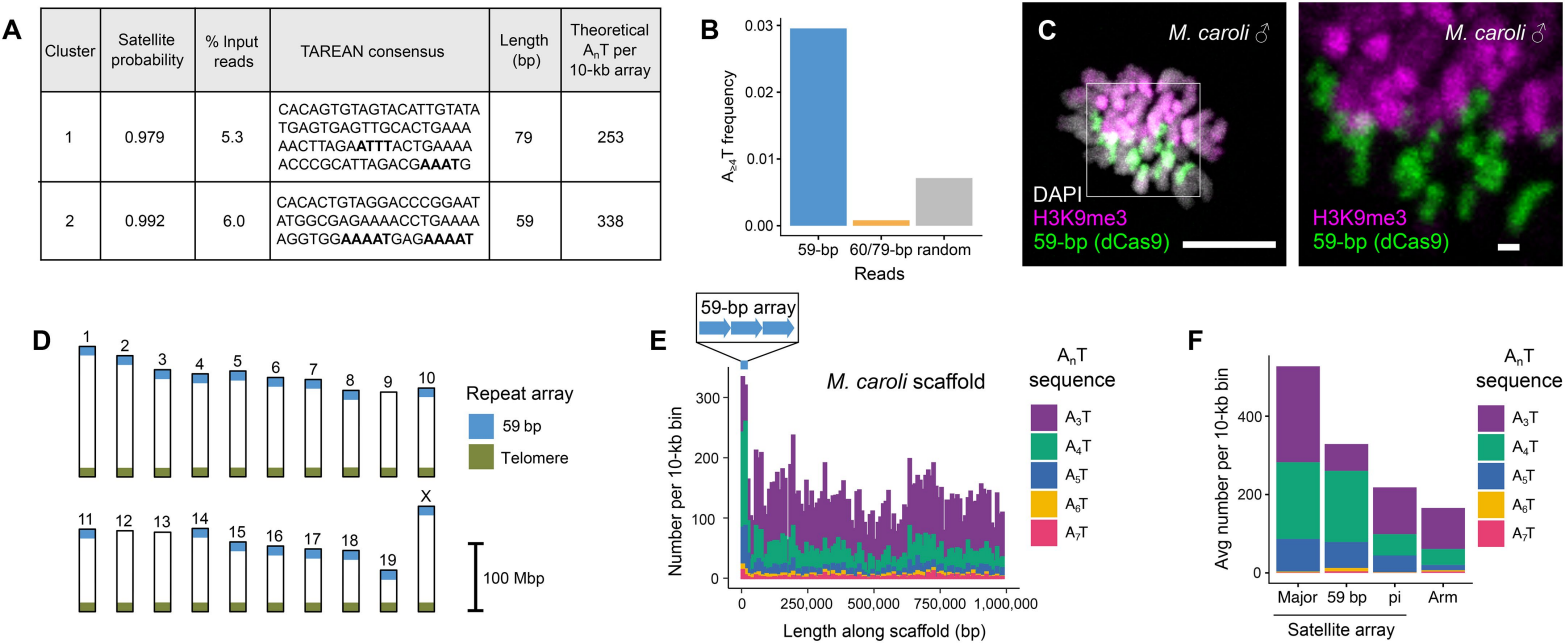
Arrays of 59-bp A_n_T-rich satellites are present at *M. caroli* pericentromeres. (**A**) Consensus sequences and predicted A_n_T frequencies of two TAREAN read clusters predicted to be satellite arrays in *M. caroli*. (**B**) A_≥4_T frequency of *M. caroli* short reads mapped to 59-bp consensus sequences (6,738,028 reads), 60/79-bp consensus sequences (8,952,232 reads), or random reads (8,952,232 reads). (**C**) Zygotes generated with *M. caroli* sperm expressing dCas9:mCherry (green) with gRNAs targeting the 59-bp satellite. Embryos were arrested in mitosis by APC/C inhibition (proTAME) and then fixed and stained for H3K9me3 (magenta) to mark maternal *M. musculus* chromosomes and DAPI (gray). Representative image is a max intensity z-projection. Scale bars, 10 or 1 μm (inset) (*N* = 2). (**D**) Schematic showing the location of 59-bp and telomere repeat arrays on the 20 chromosomal scaffolds of our *M. caroli* long-read assembly. Repeat array sizes are not to scale. (**E**) Histogram showing the number of various A_n_T sequences per 10-kb bin along the first megabase of *M. caroli* chromosome 16. The position of the 59-bp array is indicated by the horizontal blue bar. (**F**) The average number of various A_n_T sequences per 10-kb bin in major, 59-bp, and pi satellite arrays as well as *M. caroli* chromosome arms.

Using an established computational pipeline (TAREAN) (*50*) with published *M. caroli* short-read sequences (see Materials and Methods), we identified two read clusters with a high probability of being satellites. The first cluster makes up 5.3% of input reads, with a consensus sequence identical to the known 79-bp satellite (Fig. 4A) (*49*). Reads corresponding to both the 60-bp and 79-bp known satellites are grouped within this cluster, likely reflecting their high sequence similarity (*49*) (fig. S4, A, B, and E, and table S3). The second cluster makes up 6% of input reads, with a 59-bp consensus sequence (Fig. 4A) that includes two A_4_T runs and is more similar to *M. musculus* major satellite subrepeats than to the *M. caroli* 60-bp and 79-bp satellites (fig. S4D). Arrays of the 59-bp consensus satellite would have a frequency of A_≥4_T sequences similar to major satellite arrays (i.e., 338 versus ~280 per 10 kb) (Fig. 4A and table S2). In contrast to the 60-bp/79-bp cluster, however, the 59-bp cluster includes many variants, suggesting a relatively diverse satellite family (fig. S4C and table S4). To capture this sequence diversity in our analysis, we quantified A_≥4_T in reads mapped to a concatenated set of 59-bp satellite variants outputted by TAREAN (see Materials and Methods). The observed A_≥4_T frequency (0.030) closely matches the predicted value from the consensus (0.034) (Fig. 4B) and is 4.3-fold higher than a random sampling of reads (0.007), similar to the enrichment of A_≥4_T sequences in major satellite compared to chromosome arms (Fig. 3D and table S2). Furthermore, reads mapped to a concatenated 60-bp/79-bp sequence show a very low A_≥4_T frequency (0.0008), consistent with the consensus sequence of both satellites not encoding any A_≥4_Ts. Together, these results reveal a 59-bp satellite family in *M. caroli* with a frequency of longer A_n_T sequences similar to major satellite.

The 79-bp and 60-bp *M. caroli* satellites have hallmarks of centromeric satellites, such as high sequence homogeneity (*1*, *51*) based on k-mer analysis (fig. S4B and table S3) and CENP-B box motifs in the 79-bp satellite (*49*). We therefore hypothesized that these two satellites underlie centromeres, whereas the relatively diverse 59-bp satellites primarily make up pericentromeres. To visualize 59-bp satellite arrays on *M. caroli* chromosomes, we injected *M. musculus*/*M. caroli* hybrid zygotes with mRNA encoding dCas9:mCherry and a cocktail of guide RNAs (gRNAs) designed against reads within the 59-bp satellite cluster (fig. S4E) (see Materials and Methods). To ensure the specificity of our gRNAs, protospacer adjacent motif (PAM) sites are present in 59-bp satellites but absent in both the 60-bp and 79-bp satellites (fig. S4E). We find that dCas9:mCherry localizes to paternal *M. caroli* metaphase chromosomes but not to maternal *M. musculus* pericentromeres, despite the sequence similarity between the 59-bp satellite and *M. musculus* major satellite (Fig. 4C), showing the specificity of our gRNAs. Moreover, mCherry signals are located near centromeric ends of *M. caroli* chromosomes, consistent with our prediction that the 59-bp satellite is pericentromeric.

As an independent approach to localizing 59-bp satellite arrays on *M. caroli* chromosomes, we generated a de novo *M. caroli* genome assembly from long PacBio HiFi and Hi-C reads. Our assembly includes 20 chromosome-sized scaffolds, each aligning well to one of the 20 chromosomes of a previously published short-read *M. caroli* assembly (CAROLI_EiJ_v1.1) (fig. S5A), indicating that we have assembled all chromosome arms. Our assembly contains complete copies of 99.6% of 9226 universal mammalian single-copy protein-coding genes (fig. S5B), indicating that it is relatively complete in terms of gene content. Almost all chromosome-sized scaffolds begin with a short 59-bp satellite array, and all end with a short telomere array (Fig. 4D and fig. S5C). Their positions at the ends of chromosome-sized scaffolds show that 59-bp satellites are likely pericentromeric. The 59-bp satellites can be interrupted by other sequences, resulting in multiple discrete arrays that range in size from 400 bp to 15 kb (fig. S5D). Because these small arrays fall within the average length of a PacBio read (~15 kb), it suggests that they are assembled by a few long reads anchoring them to unique sequence in chromosome arms. In contrast, most of the 59-bp satellite DNA (predicted by TAREAN to comprise 6% of the *M. caroli* genome; Fig. 4A) are likely largely unassembled. Consistent with this inference, 60-bp and 79-bp satellite arrays are not present in these chromosome-sized scaffolds and likely reside in unassembled centromeres.

We quantified A_n_T frequencies in the assembled 59-bp arrays and found that they are similar to predictions from the consensus sequence (Fig. 4E). We directly compared the average number of A_n_T sequences per 10 kb between the different satellite arrays and find that both major satellite and 59-bp arrays have a higher enrichment of A_≥4_T sequences compared to chromosome arms (4.6-fold and 4.3-fold, respectively) than pi satellite arrays (1.6-fold) (Fig. 4F). Overall, our analyses of four species’ satellite sequences combined with their differing abilities to recruit PRC1 indicate that longer A_n_T sequences drive formation of PRC1 heterochromatin at paternal pericentromeres in the first mitosis.

### Consequences of PRC1 heterochromatin for pericentromere function

**Fig. 5.**
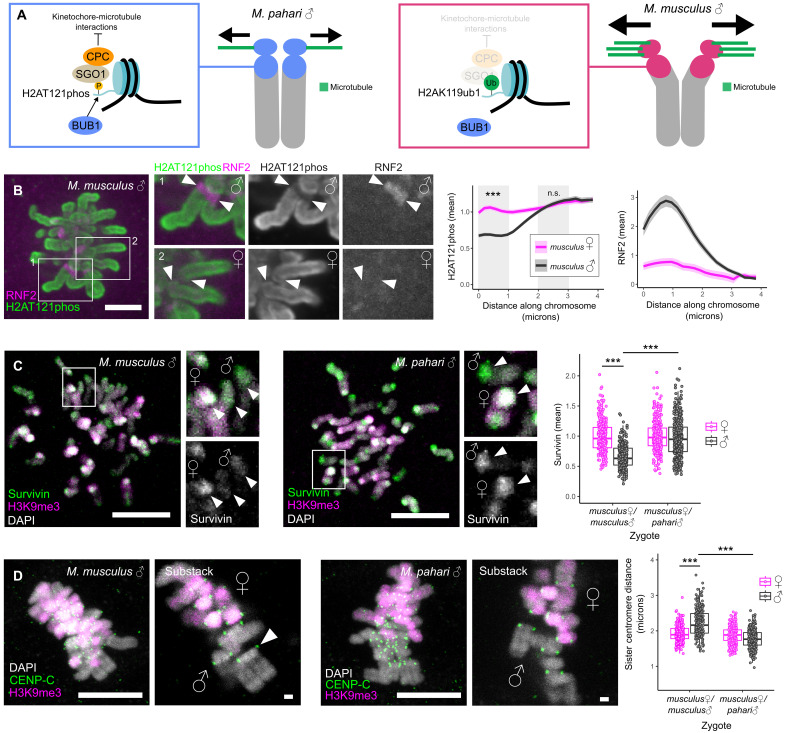
PRC1 pericentric heterochromatin inhibits CPC recruitment. (**A**) Schematic shows how H2AK119ub1 can inhibit H2AT121phos and CPC recruitment, resulting in increased distance between metaphase sister kinetochores at paternal *M. musculus* pericentromeres (right) versus *M. pahari* pericentromeres (left). (**B**) Pure *M. musculus* zygotes were arrested at metaphase by APC/C inhibition (proTAME) and then fixed and stained for RNF2 (PRC1 subunit, magenta) and H2AT121phos (green). Top and bottom insets show paternal (RNF2 positive) and maternal (RNF2 negative) chromosomes, respectively; arrowheads point to pericentromeric ends of chromosomes. Graphs show average H2AT121phos and RNF2 intensity along maternal (*n* = 56) and paternal (*n* = 54) chromosomes, starting from pericentric ends. Statistical differences between maternal and paternal chromosomes from 0 to 1 and 2 to 3 microns along their length (gray boxes) were calculated (*N* = 2). (**C**) Zygotes generated with *M. musculus* or *M. pahari* sperm were arrested in mitosis with a kinesin-5 inhibitor (STLC) and then fixed and stained for Survivin (green) and H3K9me3 (magenta). Arrowheads in insets point to pericentric Survivin staining. Graph shows the mean Survivin intensity of maternal and paternal pericentromeres in both types of zygotes. Each point represents a single pericentromere (*n* = 253 to 374 for each group, *N* = 3), and boxes represent interquartile ranges. (**D**) Zygotes generated with *M. musculus* or *M. pahari* sperm were arrested at metaphase by APC/C inhibition (proTAME) and then fixed and stained for CENP-C (green) and H3K9me3 (magenta). Arrowhead points to a pair of paternal *M. musculus* sister kinetochores. Graph shows distances between sister kinetochores for maternal and paternal chromosomes in both types of zygotes. Each point represents a single pair (*n* = 230 to 284 for each group, *N* = 3). *P* values were calculated by a Kruskal-Wallis test followed by Dunn’s test using Bonferroni correction for multiple testing (***P < 0.001). Images are max intensity z-projections. Scale bars, 10 or 1 μm (insets). n.s., not significant.

Next, we asked whether the difference in PRC1 heterochromatin between paternal *M. musculus* and *M. pahari* chromosomes has consequences for pericentromere function. Notably, H2AK119ub1 is positioned close to the site of another posttranslational modification: phosphorylation of H2AT121 by Bub1 kinase. H2AT121phos recruits the CPC to pericentromeres via Sgo1 (Fig. 5A) to ensure accurate chromosome segregation by regulating kinetochore-microtubule interactions during mitosis (*14*–*16*). Chromatin immunoprecipitation sequencing (ChIP-seq) and in vitro assays on reconstituted nucleosomes have shown that H2AK119ub1 and H2AT121phos are mutually exclusive (*52*), suggesting that one modification physically blocks the deposition of the other on the same H2A C-terminal tail. We hypothesized that H2AK119ub1 occludes Bub1 kinase from phosphorylating H2AT121 and thereby reduces CPC binding. This hypothesis predicts low H2AT121phos and low CPC on paternal *M. musculus* pericentromeres (Fig. 5A), which have high H2AK119ub1 compared to maternal *M. musculus* and paternal *M. pahari* pericentromeres (Fig. 1, C and E).

To test this prediction, we first visualized H2AT121phos, the CPC subunit Survivin, and the PRC1 subunit RNF2. In *M. musculus*♀/*M. musculus*♂ zygotes arrested at metaphase by chemical inhibition of the anaphase-promoting complex/cyclosome (APC/C), we found that H2AT121phos is present along the entire chromosome (Fig. 5B) as reported in the previous cell cycle in mouse oocytes (*53*, *54*). Moreover, we found an inverse relationship between RNF2 and H2AT121phos, with the presence of RNF2 at paternal pericentromeres coinciding with a reduction in H2AT121phos (Fig. 5B). In contrast, maternal pericentromeres with undetectable RNF2 show no reduction in H2AT121phos. Consistently, when we compared regions of maternal and paternal chromosomes that both lack RNF2, H2AT121phos intensities were similar, indicating that reduced H2AT121phos depends on RNF2. On average, H2AT121phos is reduced to ~60% at paternal *M. musculus* pericentromeres compared to maternal pericentromeres (Fig. 5B). Consistent with H2AK119ub1 heterochromatin inhibiting H2AT121phos, Survivin intensity on paternal *M. musculus* pericentromeres is reduced to ~65% compared to paternal *M. pahari* and maternal *M. musculus* pericentromeres, both of which form relatively less H2AK119ub1 (Fig. 5C). Survivin is also reduced on paternal *M. caroli* pericentromeres, which have H2AK119ub1, compared to maternal *M. musculus* pericentromeres (fig. S6). Together, these results show that PRC1 heterochromatin on paternal pericentromeres inhibits the acquisition of H2AT121phos and thereby reduces CPC recruitment.

The Aurora B kinase subunit of the CPC phosphorylates kinetochore proteins to destabilize kinetochore-microtubule interactions (*55*). In tissue culture cells, expression of a nonphosphorylatable mutant of a key Aurora B substrate, Hec1, results in increased stabilization of kinetochore-microtubule interactions and increased forces pulling the sister kinetochores apart at metaphase (*56*). Increased interkinetochore distance therefore provides a functional readout for reduced Aurora B activity (Fig. 5A). To test for functional consequences of CPC differences between pericentromeres in the zygote, we measured distances between sister kinetochores at metaphase. We found increased interkinetochore distances for paternal *M. musculus* chromosomes compared to either maternal *M. musculus* or paternal *M. pahari* chromosomes (Fig. 5D). This result is consistent with our hypothesis that PRC1 heterochromatin inhibits H2AT121 phosphorylation, down-regulates the CPC, and stabilizes kinetochore-microtubule interactions on paternal *M. musculus* chromosomes. Increased interkinetochore distances might also reflect reduced pericentric cohesion because, in addition to recruiting the CPC, Sgo1 also recruits phosphatases that counteract cohesin removal that occurs along chromosome arms during mitotic prophase (Fig. 5A) (*57*, *58*). However, sister chromatids appear in close proximity along most of their lengths during zygotic mitosis (Fig. 5D) (*59*), suggesting that cohesins are not removed during prophase at this stage of development. Therefore, we interpret the increase in interkinetochore distance as a consequence of reduced CPC activity.

**Fig. 6.**
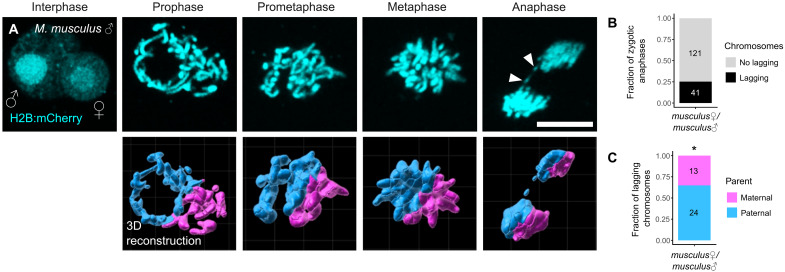
Paternal *M. musculus* chromosomes are more prone to segregation errors than maternal *M. musculus* chromosomes. (**A**) Live imaging snapshots show a *M. musculus* ♀/*M. musculus* ♂ zygote expressing H2B:mCherry (cyan) progressing through the first mitosis (top), with arrowheads indicating lagging chromosomes in anaphase, and a 3D reconstruction of maternal (magenta) and paternal (blue) chromosomes (bottom). (**B** and **C**) Plots showing the fraction of zygotes with lagging chromosomes in anaphase (B) and the proportion of maternal and paternal chromosomes contributing to these lagging events (C). Raw numbers are shown within each bar (*N* = 3). *P* value was calculated using a one-sided binomial test to assess whether paternal chromosomes lagged more often than the null expectation of 50% (*P < 0.05). Images are max intensity z-projections. Scale bar, 10 μm.

The known function of the CPC in regulating kinetochore-microtubule interactions suggests that the observed differences between maternal and paternal pericentromeres may lead to differences in the frequency of chromosome segregation errors in anaphase (e.g., due to a kinetochore connected to microtubules emanating from both spindle poles) (*60*). To test this possibility, we performed live imaging of pure-species *M. musculus* zygotes, in which paternal pericentromeres form PRC1 heterochromatin and recruit less CPC than maternal pericentromeres. The paternal pronucleus is larger than the maternal pronucleus in late G_2_, allowing us to distinguish and automatically track paternal and maternal chromosomes during the first mitosis (Fig. 6A and movies S1 and S2). We observed at least one lagging chromosome in ~25% of anaphases (41 of 162) (Fig. 6B). In most cases (37 of 41), we were able to determine the parental origin and found that paternal chromosomes are overrepresented (24 of 37 = 65%) compared to the null prediction of 50% (*P =* 0.049) (Fig. 6C). This result indicates that paternal chromosomes are more prone to segregation errors in *M. musculus* zygotes, consistent with PRC1-enriched pericentric heterochromatin compromising chromosome segregation fidelity.

## DISCUSSION

Long-read sequencing technology continues to reveal the extensive diversity of satellite DNA sequence composition (*8*, *9*, *11*, *61*), but experimental systems that test the functional impact of this diversity remain limited. In the mouse model species *M. musculus*, centromeric and pericentromeric satellite variation is limited to copy number differences of the same satellite sequences (*2*, *5*, *53*). Our hybrid embryo system overcomes this limitation by incorporating sequence variation between satellites that is only found in more divergent nonmodel mouse species.

Using this system, we demonstrated that *M. musculus* major satellite sequence is more effective than *M. pahari* pi satellite sequence at recruiting PRC1 during mitosis. Consistent with the known sequence preferences of AT-hook proteins (*29*), AlphaFold3 modeling indicates that this difference results from the AT-hook of CBX2*^musculus^* preferentially binding the exceptionally narrow minor groove of long A_n_T sequences, which are enriched in *M. musculus* major satellite arrays when compared to *M. pahari* pi satellite arrays and chromosome arms. Furthermore, our satellite DNA-based model of PRC1 recruitment accurately predicted a previously unidentified A_n_T-rich pericentric satellite (59-bp satellite) in *M. caroli* as well as reduced PRC1 recruitment to A_n_T-depleted minor satellites in *M. spretus.* Together, these results indicate that the enrichment of narrow minor grooves generated by long A_n_T sequences largely dictates PRC1 recruitment during mitosis, although other species-specific sperm features (e.g., chromatin packaging, histone variants, and RNAs) could also contribute.

Our finding of divergence between *Mus* species in the ability of pericentric satellites to recruit PRC1 during mitosis is unexpected, given the importance of PRC1 in maintaining *M. musculus* paternal pericentromere integrity during the first zygotic anaphase (*24*). We propose that PRC1 localization to *M. pahari* pericentromeres during interphase, rather than its persistence on mitotic chromosomes, is sufficient for pi satellite array stability during mitosis. Consistent with this possibility, CBX2 recruits DAXX and promotes H3.3 deposition at paternal pericentromeres during zygotic interphase (*24*). PRC1 silencing of satellite transcription also occurs during interphase (*20*). This model is consistent with our observation that all tested species establish H3K27me3 at paternal pericentromeres, suggesting that PRC1 enrichment during zygotic interphase through the chromodomain of CBX2 is conserved in *Mus.* Alternatively, *M. musculus* major satellites and *M. caroli* 59-bp satellites may have specific properties that require their packaging in PRC1 heterochromatin during mitosis, whereas *M. pahari* pi satellites can rely on PRC2-mediated H3K27me3.

As another alternative model, the *M. pahari* egg cytoplasm may have specific adaptations that allow PRC1 to be recruited to mitotic pericentromeres by a different mechanism. Because the core CBX2 AT-hook sequence is conserved across Muridae (fig. S7), it is unlikely that the AT-hook of CBX2*^pahari^* has adapted to bind A/T sequences enriched at *M. pahari* pi satellites. Similarly, the CBX2 chromodomain is identical across Muridae, indicating that the chromodomain of CBX2 *^pahari^* has not adapted to have a higher binding affinity toward H3K27me3. However, vertebrates encode multiple PRC1 complexes that share the core ubiquitin ligase subunits (RING1A/1B) but differ in associated proteins that diversify PRC1 function, including how the complex is recruited to chromatin (*62*). Thus, another chromatin targeting subunit expressed in *M. pahari* zygotes might recognize paternal *M. pahari* pericentromeres during mitosis.

PRC1 localization to *M. pahari* paternal pericentromeres during G_2_ but not mitosis suggests that binding of the CBX2 chromodomain to H3K27me3 is cell cycle dependent. We speculate that Aurora B–mediated phosphorylation of H3S28 during mitosis (*38*) prevents the CBX2 chromodomain from binding H3K27me3, leading to loss of PRC1 from *M. pahari* pericentromeres, which lack A_n_T-rich satellites. Cytosolic deubiquitinating enzymes would then remove H2AK119ub1 after nuclear envelope breakdown (*63*). This removal, together with our finding that H2AK119ub1 inhibits CPC recruitment, provides a mechanism for pericentric satellites to affect paternal chromosome segregation during the first zygotic mitosis. A_n_T-rich satellites at paternal *M. musculus* and *M. caroli* pericentromeres maintain PRC1 in mitosis through the CBX2 AT-hook, leading to low CPC. In contrast, low PRC1 at paternal *M. pahari* pericentromeres leads to high CPC, similar to maternal *M. musculus* pericentromeres, where H3K9me3-based heterochromatin inhibits PRC1 recruitment (*20*, *22*).

Two lines of evidence support the functional significance of these differences in mitotic PRC1. First, low CPC is expected to stabilize kinetochore microtubules, leading to increased sister kinetochore separation as we observe during the first zygotic metaphase for paternal *M. musculus* chromosomes (high PRC1) compared to maternal *M. musculus* or paternal *M. pahari* chromosomes (low PRC1). Looser chromatin packing due to CBX2/PRC1 retention on mitotic chromosomes could also contribute to the increased sister kinetochore separation. Second, we find that paternal *M. musculus* chromosomes lag significantly more than maternal *M. musculus* chromosomes during zygotic anaphase, indicating more frequent kinetochore-microtubule attachments errors with high PRC1 and low CPC.

We propose that compromised paternal chromosome segregation fidelity, due to the PRC1-mediated decrease in CPC recruitment (and/or decrease in chromatin compaction), selects against large A_n_T-rich satellite arrays in natural populations. Consistent with this idea, wild-caught *M. musculus* have roughly 10 times fewer major satellite repeats than inbred laboratory strains, where natural selection is relatively weak (*5*). However, pericentric PRC1 heterochromatin is required for the transcriptional silencing and stability of paternal major satellite arrays (*22*, *24*), suggesting an evolutionary trade-off between proper packaging of major satellites and chromosome segregation fidelity.

To avoid this trade-off in most cell cycles, we propose that H3K9me3 heterochromatin serves to prevent DNA sequence–based pathways of PRC1 recruitment, preserving pericentric CPC during mitosis and chromosome segregation fidelity. This epigenetic heterochromatin suppresses functional diversity that might otherwise arise from differences in satellite DNA sequence composition. In contrast, our findings highlight the first zygotic cell cycle as a unique environment where heterochromatin formation and function are sensitive to satellite DNA sequence evolution due to the loss of H3K9me3 from the paternal genome during spermiogenesis.

## MATERIALS AND METHODS

### Mouse strains

The mouse strain representing *M. musculus* in our ICSI experiments is FVB/NJ, purchased from the Jackson Laboratory (strain no. 001800). FVB/NJ was chosen because its MII eggs can survive ICSI and because it is an inbred strain. *M. caroli* (CAROLI/EiJ, strain no. 000926) and *M. pahari* (PAHARI/EiJ, strain no. 002655) were also purchased from the Jackson Laboratory. We maintain our own colony of *M. pahari* because the Jackson Laboratory no longer carries this species. *M. spretus* was purchased from RIKEN BioResource Research Center (SPR2, RBRC00208). The mouse strain representing *M. musculus* in our live imaging experiments, CF-1, was purchased from Envigo, Inotiv, Indianapolis, IN, USA (Hsd:NSA). All animal experiments and protocols were approved by and conducted according to the regulations of the University of Pennsylvania (Institutional Animal Use and Care Committee, protocol no. 804882) or the University of Missouri (Animal Care Quality Assurance Ref. no. 9695).

### Intracytoplasmic sperm injection

To prepare sperm for ICSI, the epididymis and vas deferens were removed from males and dissected in phosphate-buffered saline (PBS) to release mature sperm. Sperm were allowed to swim-out for 10 min on a 37°C slide warmer. After swim out, sperm were pelleted by centrifugation at 700*g* at 4°C for 5 min. The PBS supernatant was removed, and sperm were then washed twice with ice-cold nuclear isolation media (NIM; 123 mM KCl, 2.6 mM NaCl, 7.8 mM Na_2_PO_4,_ 1.4 mM KH_2_PO_4_, and 3 mM EDTA, pH adjusted to 7.2 using 1 M KOH) with 1% polyvinyl alcohol (PVA). After the second wash, sperm were resuspended in 100 μl of NIM 1% PVA and then sonicated using a Branson Sonic Bath Model 1210 for 15- to 20-s intervals until at most 30% of sperm had their heads detached from their tails. Sonicated sperm were washed twice with NIM 1% PVA. After the second wash, the sperm pellet was resuspended in a 1:1 solution of glycerol and NIM 1% PVA and placed at −20°C until the day of injection (the following day or 1 week later).

Females were super-ovulated by injection with 5 U of pregnant mare serum gonadotropin (PMSG; Peptides International) followed by injection with 5 U of human chorionic gonadotropin (hCG; Sigma-Aldrich) 48 hours later. MII eggs were collected from females 14 to 15 hours post-hCG injection and placed in M2 media (Sigma-Aldrich) with hyaluronidase (0.15 mg/ml) to remove cumulus cells. MII eggs were then washed through four drops of M2 media supplemented with bovine serum albumin (4 mg/ml; M2 + BSA) and left on a 37°C plate warmer until injection. Ten to 20 microliters of sonicated sperm was diluted in 200 μl of NIM 1% PVA, gently vortexed, and then washed twice with NIM 1% PVA. After the last wash, the sperm pellet was resuspended in 100 μl of NIM 1% PVA and left on ice until injection.

Batches of 10 MII eggs were injected with sperm heads at a time in room temperature drops of M2 + BSA. After injection, each batch was moved to a drop of M2 + BSA on a 37°C plate warmer and allowed to recover for 1 hour. After recovery, each batch was washed through four drops of preequilibrated Advanced K+ Simplex Optimised Media (AKSOM; MilliporeSigma) and placed in at 37°C humidified incubator with 5% CO_2_ until injections were complete. Depending on the experiment, at least 3 hours after injection, eggs were then moved into a drop of AKSOM with either 10 μM STLC (kinesin-5 inhibitor, Sigma-Aldrich) (to arrest cells in mitosis with monopolar spindles) or 5 μM proTAME (APC/C inhibitor, R&D Systems) (to arrest zygotes in metaphase) and then allowed to develop overnight in a 37°C incubator with 5% CO_2_. Zygotes arrested in mitosis were fixed in 2% paraformaldehyde 19 hours postsperm injection. To stain for RNF2 and H3K27me3 in G_2_ zygotes, injected eggs were moved to AKSOM and then allowed to develop 14 hours postsperm injection, at which point they were fixed in 2% paraformaldehyde.

### Fixing and staining

Zygotes were fixed in 37°C 2% paraformaldehyde in PBS for 20 min, washed through three pools of blocking solution (PBS containing 0.5% BSA and 0.01% Tween 20) and then left at 4°C overnight. The next morning, cells were permeabilized in PBS containing 0.5% Triton X-100 (Sigma-Aldrich) for 15 min at room temperature, quickly washed through two pools of blocking solution, and then allowed to block for 20 min at room temperature. For H3K9me3 and H3K27me3 staining, the cells were treated with lambda phosphatase [1600 U, New England Biolabs (NEB)] for 1 hour at 37°C in a humidified chamber. Phosphatase treatment is required to detect H3K9me3 and H3K27me3 staining on mitotic chromosomes, presumably by removing adjacent H3S10 and H3S28 phosphorylation (*64*). Embryos were then quickly washed through two pools of blocking solution and then incubated with primary antibodies for 1 hour in a dark humidified chamber at room temperature (or overnight at 4°C for H2AT121phos staining). Afterward, embryos underwent three 15-min washes in blocking solution and then were incubated with secondary antibodies for 1 hour in a dark humidified chamber at room temperature. Cells then underwent three 15-min washes in blocking solution and were mounted in VECTASHIELD with DAPI (Vector) to stain chromosomes. For cells that were stained with Sytox Green (Invitrogen), the first wash after incubation with secondary antibodies included 1 μM Sytox Green, and after the next two washes, cells were mounted in VECTASHIELD without DAPI. Primary antibodies used are rabbit anti-H2AK119ub1 (1:800, Cell Signaling, D27C4), rabbit anti-H3K27me3 (1:700, Cell Signaling, C36B11), mouse anti-H3K9me3 (1:200, Active Motif, 39285), rabbit anti-H3K9me3 (used when costaining against RNF2, 1:500, Active Motif, 39162), rabbit anti-Survivin (1:500, Cell Signaling, 71G4B7), rabbit anti-H2AT120phos (1:2500, Active Motif, 39392), mouse anti-RNF2/Ring1B (1:500, Active Motif, 39664), and rabbit anti–CENP-C (*65*) (1:500). Secondary antibodies used are donkey anti-rabbit Alexa Fluor 488, donkey anti-mouse Alexa Fluor 594, donkey anti-rabbit Alexa Fluor 594, and donkey anti-mouse Alex Fluor 647. All secondary antibodies are purchased from Invitrogen and used at a dilution of 1:500.

### Microscopy

Some confocal images were collected as z-stacks with 0.5-μm intervals, using a microscope (DMI4000 B; Leica) equipped with a 63× 1.3 numerical aperture glycerol-immersion objective lens, an xy piezo Z stage (Applied Scientific Instrumentation), a spinning disk confocal scanner (Yokogawa Corporation of America), an electron multiplier charge-coupled device camera (ImageEM C9100-13; Hamamatsu Photonics), and either an LMM5 (Spectral Applied Research) or Versalase (Vortran Laser Technology) laser merge module, controlled by MetaMorph software (Molecular Devices, v7.10.3.294). Confocal images were also collected using a Leica TCS SP8 Four Channel Spectral Confocal System with a 63× objective lens. A z-stack of 0.3-μm intervals was used to collect images of embryos used to calculate sister kinetochore distances. All samples in an experiment were imaged using the same laser settings.

### Image quantification and statistical analysis

All image analysis was carried out using ImageJ/Fiji (*66*). To measure signal intensities along single chromosomes, we selected chromosomes that were mostly in the *XY* plane and generated max intensities projections of them. Beginning from the pericentric end, we then used the segmented line tool to manually draw a linear region of interest (ROI; roughly the width of the chromosome) along the chromosome’s length and measured the average signal intensity per unit distance along the ROI. Average background was then subtracted from these values. To more easily compare the distributions of DAPI and Sytox Green intensity, their values were normalized to the average intensity within each cell/embryo using custom R scripts.

To measure Survivin intensity at pericentromere ends, we also selected chromosomes that were mostly in the *XY* plane and generated maximum intensity projections of them. We then use the rectangular selection tool to generate square ROIs that encompassed pericentric ends of chromosomes and measured average intensity within the ROI. Average background was then subtracted from these values. The resulting values were then normalized to maternal chromosomes in each cell/embryo using custom R scripts.

To measure the distance between sister-centromeres, we uniquely labeled each centromere and extracted its three-dimensional (3D) coordinates using 3D maxima finder and 3D Roi Manager (based on CENP-C signal). We then manually assigned sister centromeres with the help of BigDataViewer to “rotate” the image stack in cases where the spindle pole axis was not in the *XY* plane and then used custom R scripts to calculate the distance between them.

*P* values were determined using R. We performed nonparametric Kruskal-Wallis tests followed by Dunn’s test and used the Bonferroni method to correct *P* values for multiple testing. Plots were made in the ggplot2 package in R.

### Satellite sequence analysis

*M. musculus* scaffolds containing major satellite arrays were found by BLASTing the major satellite consensus sequence (*5*) on the National Center for Biotechnology (NCBI) Basic Local Alignment Search Tool (BLAST) server. These scaffolds are from a long-read *M. musculus* genome assembly generated by the Mouse Genome Project and Darwin Tree of Life Project (GenBank: GCA_947593165.1) and were downloaded from the NCBI. The pi satellite array containing scaffolds are from a published *M. pahari* long-read assembly (*9*). These scaffolds were chosen because they represent relatively complete assemblies of functional *M. pahari* centromeres and pericentromeres based on CENP-A and H3K9me3 ChIP-seq. We chose to study major and pi satellite arrays because they are the most abundant pericentric satellites and because PRC1 is known to associate with major satellite (*20*).

To quantify the frequency of A/T runs of various lengths along a scaffold, we used the matchPattern() function in the Biostrings package in R (https://bioconductor.org/packages/Biostrings). Specifically, we searched for A/T runs that were at least four nucleotides long (i.e., matched “WWWW”), with overlapping matches eventually being merged into one longer A/T run by using the reduce() function. To analyze the composition of 4W and 5W A/T runs, we used the matchPattern() function to find all possible 4W and 5W sequences and then only kept matches that overlapped with 4W or 5W A/T run lengths, respectively. To analyze the composition of 6W A/T runs, we used the 6W A/T run coordinates to extract sequences within the satellite array using the Genomic Ranges package (*67*) in R. Sequences that are reverse complements of each other are redundant and therefore were merged into one. The average number of times a particular sequence occurred per 10-kb bin within an array was calculated by dividing the total number of occurrences of a particular sequence within the array by the length of the array and then multiplying by 10,000. We defined the range of the satellite arrays for each scaffold based on coordinates from satellite consensus sequence BLAST results, and chromosome arms were defined as nonsatellite regions of the same assembled scaffolds. Last, A_n_T sequences were also found along scaffolds using the matchPattern() function. To prevent overcounting of smaller AnT sequences (e.g., overlap of AAAT and AAAAT), only the longest of overlapping A_n_T sequences were counted.

### AlphaFold3 modeling

All sequences were modeled using the AlphaFold3 webserver (https://alphafoldserver.com/). To model a single dsDNA molecule encoding two distinct A/T runs flanked by four G:C base pairs, we entered a single forward and a single reverse strand (e.g., forward: 5′GGGGAAAAGGGGAAATGGGG 3′, reverse: 5′CCCCATTTCCCCTTTTCCCC 3′). We also modeled a single peptide encoding the AT-hook of CBX2 with flanking amino acids (RKRGKR**PRGRP**RKHTVTSS, AT-hook in bold). AlphaFold3 outputs five model structures (0 to 4), with 0 scored as the best and the fourth scored as the worst. Each model was visually inspected using PyMOL (The PyMOL Molecular Graphics System, version 3.0, Schrodinger LLC). All five models for each competition assay were consistent with one another (table S1). To rule out the possibility that the relative position along the dsDNA molecule dictates where the AT-hook binds, we flipped the relative positions of the two A/T sequences along the dsDNA. For example, we modeled both 5′GGGGAAAAGGGGAAATGGGG 3′ and 5′GGGGAAATGGGGAAAAGGGG3′ sequences (fig. S2G). For each 5W model, the width of the minor groove was measured as the angstrom distance from the center of the *i* phosphate group on one strand to the *i* + 3 phosphate group on the complementary strand (black dotted lines in fig. S3) using PyMOL. A total of 5.8 Å was subtracted from all raw minor groove width measurements to account for the sum of the van der Waals radii of the two phosphate groups (*68*). An average minor groove width for each dsDNA was calculated from all five AlphaFold3 models.

### TAREAN short-read analysis

The 100-bp paired-end Illumina reads generated by the Wellcome Sanger Institute as part of the Caroli Genome Project were downloaded from the European Nucleotide Archive (ENA) (ENA Run: ERR133993) and processed using the Galaxy RepeatExplorer webserver (*69*). Read quality was assessed with FastQC. Using the “Preprocessing of FASTQ paired-end reads” tools in Galaxy, the final base of each read was trimmed due to low average quality (<Q20), reads containing ambiguous nucleotides (Ns) were removed, and the remaining read pairs were interleaved into a single FASTA file. This processed and interleaved dataset was used as input for TAREAN (*50*), which analyzed 397,722 reads with cluster merging enabled.

### Satellite DNA alignment and tree generation

Consensus sequences of *M. caroli* satellites (59-bp, 60-bp, and 79-bp) and the subrepeats of *M. musculus* satellites (minor and major) were aligned using Multiple Alignment using Fast Fourier Transform (MAFFT) using standard settings. The resulting alignment (fig. S4E) had columns with gaps removed and was used to generate a PHYLIP Neighbor Joining Tree using an F84 distance matrix model.

### Calculating A_≥4_T frequency in satellite-positive *M. caroli* short reads

The same Illumina short-read dataset described above was mapped to concatenated consensus sequences representing either the top 59-bp variants or the 60/79-bp variants using minimap2 with the -sr preset, which is optimized for short-read alignment. Only the best alignment for each read was retained. Reads mapping uniquely to the 59-bp consensus (6,738,028 reads), the 60/79-bp consensus (8,952,232 reads), and an equal number of randomly selected reads (8,952,232 reads) were extracted into separate FASTA files. To quantify A_≥4_T motif frequency, we counted all occurrences of the A_≥4_T pattern in each FASTA file using vcountPDict() function in the Biostrings package in R and divided this by the total read length in that file.

### 59-bp gRNA design, synthesis, and microinjection

To design gRNAs that capture the sequence diversity of the 59-bp satellite arrays, we used the vcountPDict() function in Biostrings to identify the most frequent 20-bp sequences located upstream of a PAM site (NGG) within reads mapping to the 59-bp TAREAN cluster. From these candidates, we selected six abundant gRNAs that do not contain PAM sites in the 60/79-bp satellites: AAATGAGAAAATCACACTGT, AAATGAGAAAATCACACTGC, AAATGAGAAAATCACACAGT, TCTCATTTTTTGACTTTTTC, TCTCATTTTCCACCTTTTTC, and TCTCATTTTTCGACTTTTTC (see fig. S4E). gRNAs were synthesized using the GeneArt Precision gRNA Synthesis Kit. A gRNA cocktail (20 ng/μl in total) and mRNA encoding dCas9:mCherry (100 ng/μl) were coinjected into MII eggs together with *M. caroli* sperm heads during ICSI. Embryos were arrested in metaphase by treatment with the APC/C inhibitor proTAME (5 μM) and then fixed and stained as described above.

### Long-read *M. caroli* genome assembly, evaluation, and repeat mapping

Flash-frozen tissue was obtained from the Jackson Laboratory from female *M. caroli* mice (CAROLI/EiJ, strain: 000926; RRID:IMSR_JAX:000926). DNA was extracted from tail tissue using the QIAGEN MagAttract HMW DNA kit according to the manufacturer’s protocol. The Pacbio SEQUEL platform was used to generate HiFi reads according to the manufacturer’s protocols. Hi-C sequencing libraries were prepared using the Arima proximity ligation method following the manufacturer’s protocols for tissue preparation. Libraries were sequenced on the Illumina NovoSeq platform. A total of 94 Gbp of HiFi and 430 Gbp of Hi-C sequencing data was generated and used for genome assembly.

HiFi reads were assembled into contigs using hifiasm (v0.25.0-r726) (*70*) with duplication-purging disabled, a recommended setting for highly inbred genomes. Scaffolding was performed using Yet another Hi-C scaffolding tool (YaHS) (*71*), which combines contigs into scaffolds by leveraging Hi-C contact frequency patterns. Hi-C reads were aligned to the primary contig assembly using bwa-mem2 (*72*) with Hi-C–specific flags (-5SP), which improve the handling of chimeric Hi-C junction reads. The resulting alignments were fed into samtools (*73*) for Binary Alignment Map (BAM) generation and processing. Mate-pair information was corrected using fixmate with the -m flag, reads were sorted by genomic coordinates, and polymerase chain reaction (PCR) and optical duplicates were removed using markdup with the -r flag. The processed Hi-C BAM and our primary assembly were used as inputs for YaHS, which was run using default parameters.

The continuity of the assembled scaffolds was evaluated using assembly-stats. The final scaffolded assembly totaled 2.84 Gb across 1194 scaffolds with a N50 of 126 Mb and 0.005% gap content (1393 gaps). Genome completeness (in terms of gene content) was assessed using BUSCO (v6.0.0) (*74*) with the mammalia_odb10 lineage dataset (fig. S5B). Most of the assembly is composed of 20 chromosome-sized scaffolds, which were mapped against a previously published *M. caroli* reference genome (CAROLI_EiJ_v1.1, Ensembl Release 115, GCA_900094665.2) (*75*) using minimap2 (*76*) with asm5 preset parameters. Alignment between the genomes was visualized using the pafr package in R to evaluate large-scale structural accuracy and identify potential scaffolding errors.

We identified the genomic locations of 59-bp and 60/79-bp satellite arrays using BLASTN with parameters optimized for short, repetitive sequences (word size = 7, dust = no). Because the 59-bp satellite family exhibits substantial sequence diversity, we used the top 11 consensus sequences generated by TAREAN’s 27-mer analysis as query sequences. In contrast, the 60/79-bp satellite family is highly homogeneous, so the single 79-bp consensus sequence produced by TAREAN was used as a query for this repeat class, which also captures the closely related 60-bp variant. Telomere arrays were detected using the matchPattern() function from the Biostrings package in R to find occurrences of four tandem telomere repeats (i.e., TTAGGGTTAGGGTTAGGGTTAGGG).

### Live imaging of *M. musculus* zygotes

#### Superovulation, collection, and in vitro fertilization of mouse eggs

CF-1 female mice aged 8 to 12 weeks were superovulated by intraperitoneal injection of 7.5 IU of PMSG (Medix Biochemica) followed 48 hours later by 7.5 IU hCG (Prospec) injection. Male mice were euthanized, and their epididymides were carefully dissected and transferred to a dish containing 100 μl of human tubal fluid (HTF) medium (MiliporeSigma, MR-070-D), overlaid with mineral oil (FujiFilm Biosciences, 9305). Sperm were collected and incubated in HTF for 1 hour at 37°C in a humidified atmosphere containing 5% CO_2_ for capacitation. Female mice were euthanized 15 to 17 hours after hCG injection, and their oviducts were dissected and transferred into a fertilization dish containing 200 μl of HTF overlaid with mineral oil. Oviducts were torn using a 27G hypodermic needle, and cumulus oocyte complexes were collected and placed in HTF. In vitro fertilization (IVF) was performed by adding 2 to 3 μl of capacitated sperm to the fertilization drop. After 3 hours of IVF culture, zygotes were washed through six drops of K+ Simplex Optimised Medium (KSOM; MiliporeSigma, MR-101).

#### Cloning, in vitro cRNA synthesis, and microinjection

The H2B:mCherry construct was linearized using Nde I (New England Biolabs). After DNA linearization, the digests were purified (QIAGEN, QIAquick PCR Purification), and in vitro transcription was carried out using the mMessage mMachine T7 kit (Ambion) according to the manufacturer’s instructions. Finally, the complementary RNA (cRNA) was purified using an RNAEasy kit (QIAGEN) and stored at −80°C. Zygotes were microinjected 4 hours postinsemination with 5 pl of the cRNA using Eppendorf FemtoJet 4i. Advanced KSOM was used as the microinjection medium. The injected zygotes were allowed to recover for 2 hours at 37°C in a humidified atmosphere containing 5% CO_2_ to allow for cRNA expression before live imaging. Microtubules were labeled in live embryos using SiR-tubulin (Cytoskeleton, CY-SC002), added to the culture medium at a final concentration of 100 nM. Microtubule images are not shown in Fig. 5E.

#### Confocal microscopy

For live-cell imaging, zygotes were transferred to small drops of KSOM medium under mineral oil in glass-bottom dishes (MatTek). Dishes were placed in a microenvironmental chamber (UNO-T-H-CO2, Okolab) and kept at 37°C in a humidified atmosphere containing 5% CO_2_. Images were acquired using a Leica Stellaris 5 confocal microscope using a 40× oil-immersion objective. Z-stacks were acquired every 8 min covering a 70-μm range (5-μm step size) for a total duration of 8 hours.

#### Data analysis

Zygotes were analyzed using the National Institutes of Health (NIH) ImageJ software (NIH, Bethesda, MD, USA) and Imaris software (Oxford Instruments, Buckinghamshire, UK). Paternal and maternal chromosomes were distinguished on the basis of the larger size of the paternal pronucleus (*77*). 3D reconstruction and manual object classification in Imaris were used to differentially label paternal and maternal chromosomes at nuclear envelope breakdown, and chromosomes were then automatically tracked using Imaris. Lagging chromosomes were identified as chromosomes remaining near the spindle equator after anaphase onset.
