# Supplementary material for: Satellite DNA sequence dictates pericentromere heterochromatin formation and function
Source: Sci Adv. 2026 Jul 15;12(29):eady2267. doi: 10.1126/sciadv.ady2267 (PMC13371928; doi:10.1126/sciadv.ady2267)
Supplement: Supplementary file 1 — Figs. S1 to S7 Legends for tables S1 to S4 Legends for movies S1 and S2 [file sciadv.ady2267_sm.pdf]

Science Advances
AAAS

Supplementary Materials for

Satellite DNA sequence dictates pericentromere heterochromatin formation and function

Piero Lamelza *et al.*

Corresponding author: Michael A. Lampson, lampson@sas.upenn.edu

Sci. Adv. **12**, eady2267 (2026)
DOI: 10.1126/sciadv.ady2267

The PDF file includes:

Figs. S1 to S7
Legends for tables S1 to S4
Legends for movies S1 and S2

Other Supplementary Material for this manuscript includes the following:

Tables S1 to S4
Movies S1 and S2

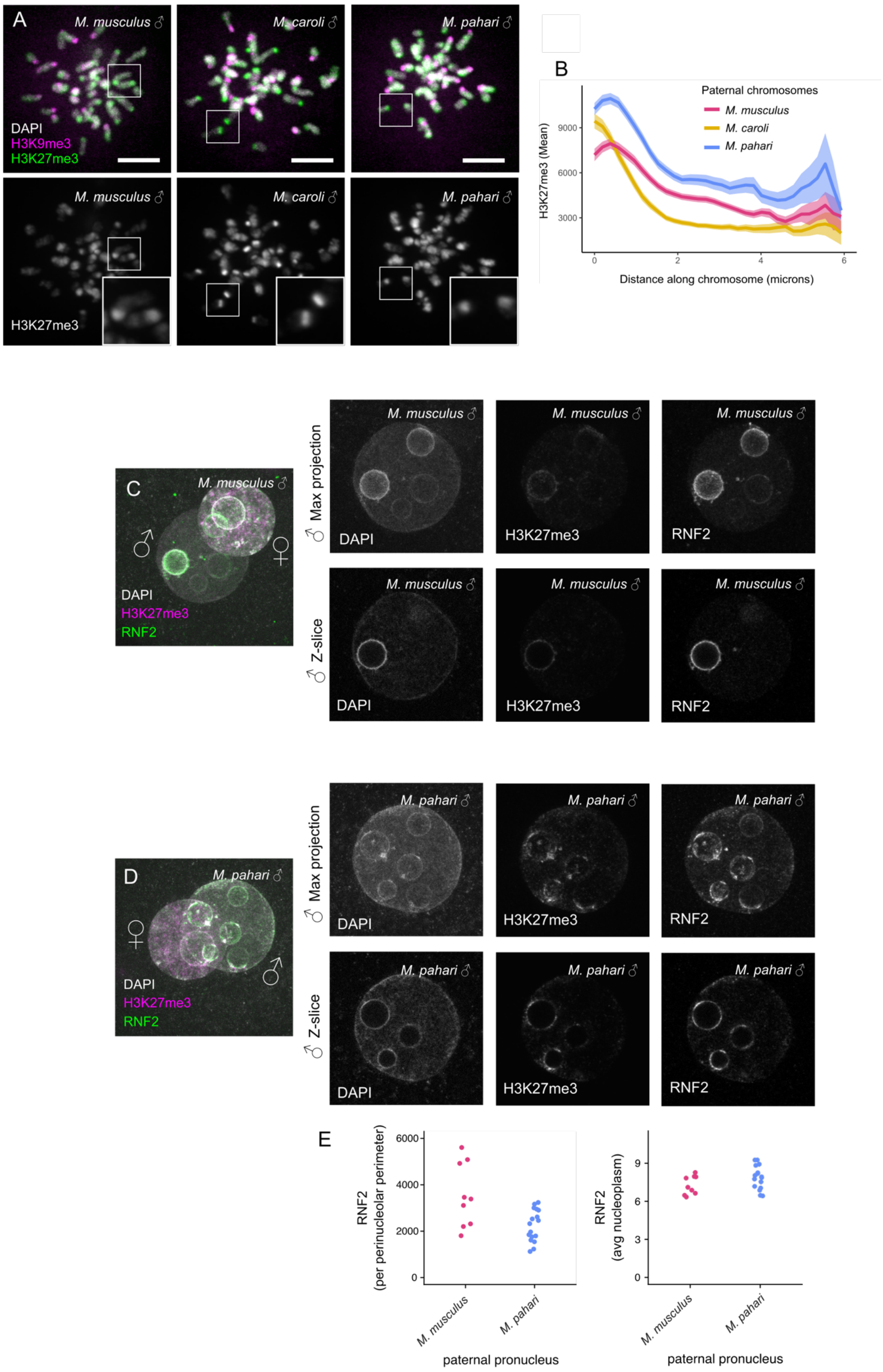
A
M. musculus ♂
M. caroli ♂
M. pahari ♂
DAPI
H3K9me3
H3K27me3
B
Paternal chromosomes
M. musculus
M. caroli
M. pahari
H3K27me3 (Mean)
9000
6000
3000
0
2
4
6
Distance along chromosome (microns)
C
DAPI
H3K27me3
RNF2
Max projection
Z-slice
D
E
RNF2 (per perinucleolar perimeter)
6000
4000
2000
RNF2 (avg nucleoplasm)
9
6
3
paternal pronucleus

Fig. S1. Paternal *M. pahari* pericentromeres recruit PRC1 during zygotic G2. **(A)** Zygotes generated with sperm from the indicated species were arrested in mitosis using a kinesin-5 inhibitor, then fixed and stained for H3K27me3 (green), H3K9me3 (magenta) to mark maternal *M. musculus* chromosomes, and DAPI (gray). **(B)** Graphs plot average H3K27me3 intensities along *M. musculus* (red, n=99), *M. caroli* (yellow, n=135) and *M. pahari* (blue, n=130) paternal chromosomes, starting from pericentric ends. S.E.M. is indicated by light band surrounding the mean line (*N*=2). **(C-D)** Zygotes in late G2 generated with *M. musculus* (C) or *M. pahari* (D) sperm were fixed and stained for H3K27me3 (magenta), RNF2 (green) and DAPI (gray). Maternal and paternal pronuclei indicated by sex symbol. Max intensity projections of whole paternal pronuclei (top row) and single Z-slices of the same paternal pronuclei (bottom row) are shown. Paternal satellites are organized around the periphery of nucleolar precursor bodies (circles without DAPI staining) (*20*, *22*, *24*). **(E)** Graphs plot either average RNF2 intensity per perinucleolar perimeter (left plot) or average nucleoplasmic RNF2 intensity (right plot) in either *M. musculus* (red=9) or *M. pahari* (blue=17) paternal pronuclei (*N*=2).

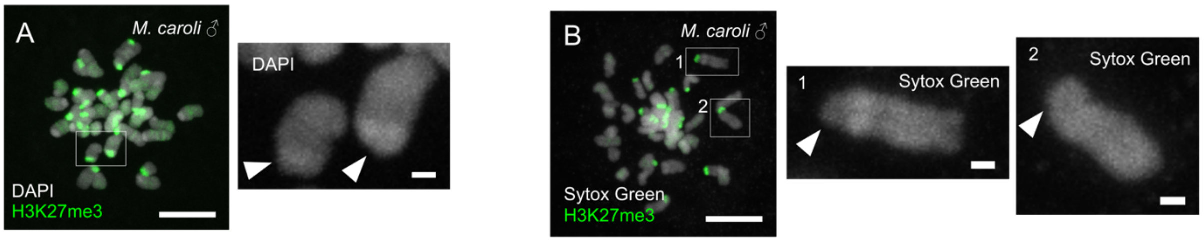

C

major satellite consensus

GAG**AAAT**GCACACTGAAGGACCTGG**AATAT**GGCGAG**AAAA**CTG**AAAAT**CACGG**AAAAT**GAG**A**
AATACACAC**TTTA**GGACGTG**AAATAT**GGCGAGG**AAAA**CTG**AAAAA**GGTGG**AAAATTT**AG**AAA**
TGTCCACTGTAGGACGTGG**AATAT**GGCAAG**AAAA**CTG**AAAAT**CATGG**AAAAT**GAGAAACATC
CACTTGACGACTTG**AAAAAT**GACG**AAAT**CAC**TAAAAAA**CGTG**AAAAAT**

pi satellite consensus

CATGATTCACTCTG**TTTTT**CATGAG**TTTT**GTGTG**TAAAA**CAAGTG**AATTT**C**TTAAA**GATC**TA**
TTAGACACATTGTAGAG**ATTTTT**GTAGAACAAGC**ATAT**G**AATAT**GAG**TTTA**GTTCC**TAAAAT**
ACTGG**TTATT**CTATG**AAAA**CATTCCAC**AATT**CTTGTTCAGAGC**AATAA**GTACAACTATCTGC
ATT

A/T run	Number per Major satellite consensus sequence	Predicted number per 10kb in Major satellite array	Number per Pi satellite consensus sequence	Predicted number per 10kb in Pi satellite array
4W	7	299	5	265
5W	8	342	8	423
6W	3	128	1	53
7W	2	85	1	53
8W	0	0	0	0
9W	0	0	0	0
≥10W	0	0	0	0
Total	20	855	15	794

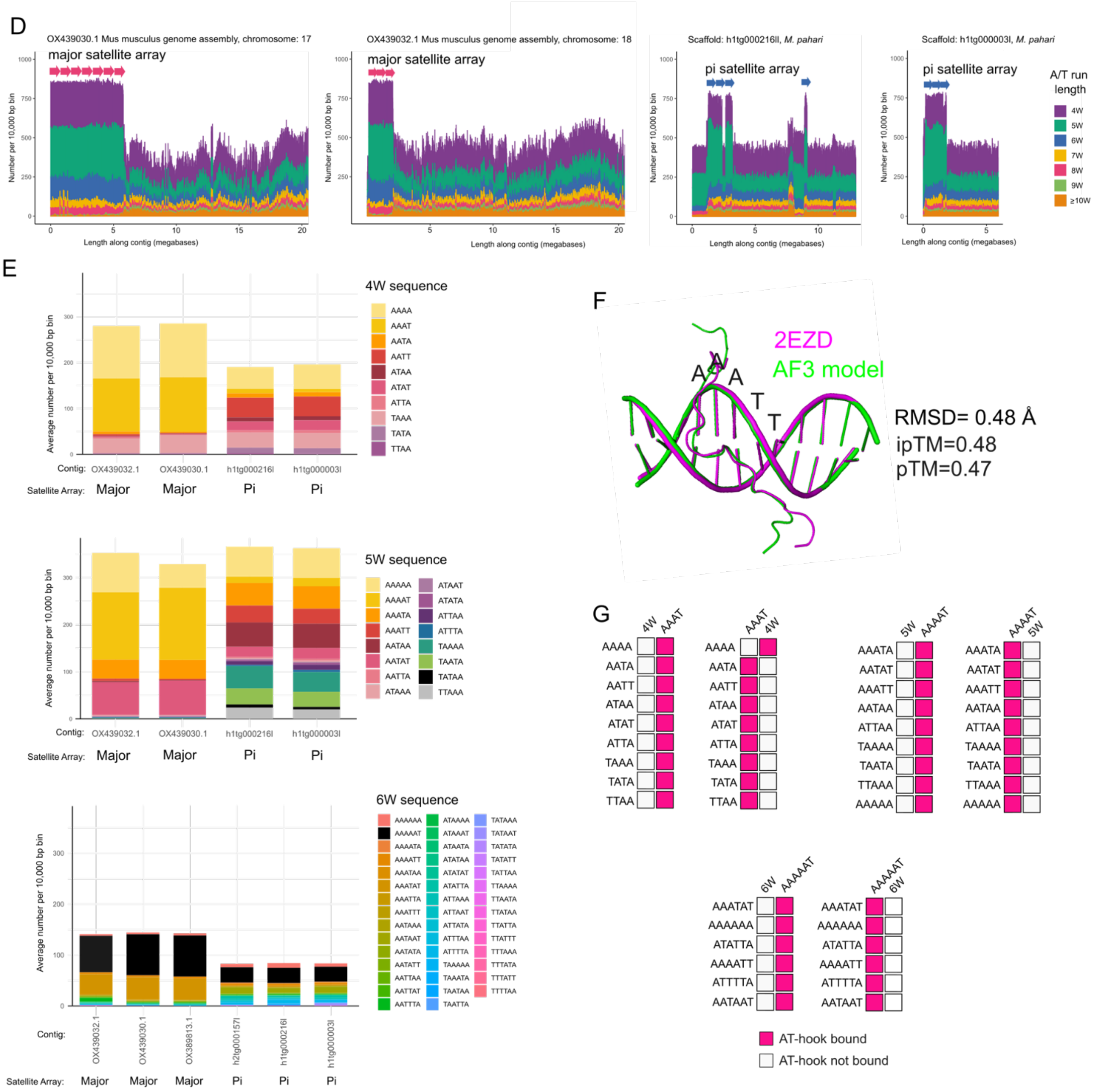

H

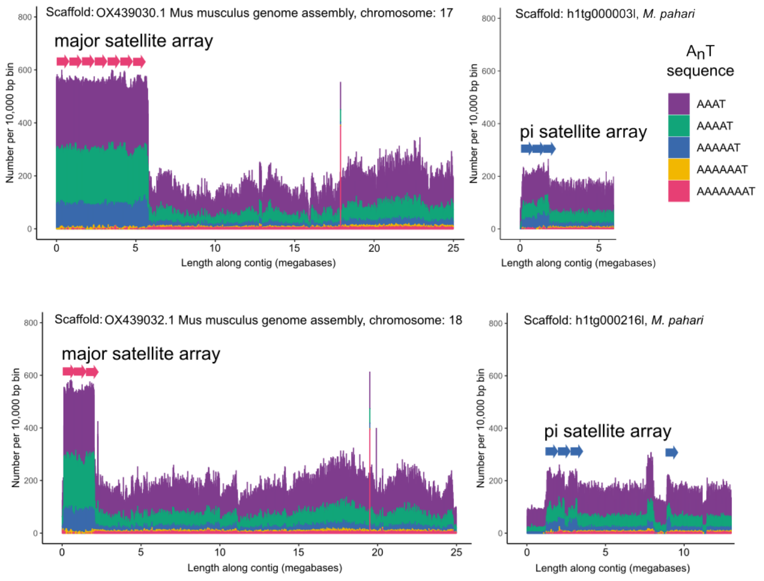

Fig. S2. Satellite sequence determinants of PRC1 binding. (A-B) Zygotes generated with *M. caroli* sperm were arrested in mitosis with a kinesin-5 inhibitor (STLC), then fixed and stained for H3K27me3 (green) to mark paternal pericentromeres and either DAPI (A, gray) or Sytox Green (B, gray). Arrowheads in insets point at paternal *M. caroli* pericentromeres. Images are max intensity z-projections; scale bars 10 µm or 1 µm (insets). **(C)** Major and pi satellite consensus sequences (left) with A/T runs greater than or equal to four shown in bold. Table (right) summarizes the number of specific A/T run lengths (4W to ≥10W) in satellite consensus sequences and their predicted frequency in satellite arrays based on consensus sequences. **(D)** Histograms plotting the number of various A/T run lengths per 10 kb bin along portions of additional genomic scaffolds containing either *M. musculus* major satellite or *M. pahari* pi satellite arrays, indicated by tandem arrows above plots. **(E)** The average number per 10 kb bin of all possible 4W and 5W and all present 6W sequences within the major and pi satellites arrays shown in Figure S2D and Figure 3A. **(F)** Alignment of the 2EZD structure (magenta) and the corresponding AlphaFold3 prediction (green) with nucleotides noted along the DNA backbone. **(G)** Summary of results of the *in silico* competitive binding assay, including when A/T sequence orders are reversed along the dsDNA. Includes results from Figure 3C. **(H)** Histograms plotting the number of various A_nT sequences per 10 kb bin along the same genomic contigs as in panel D.

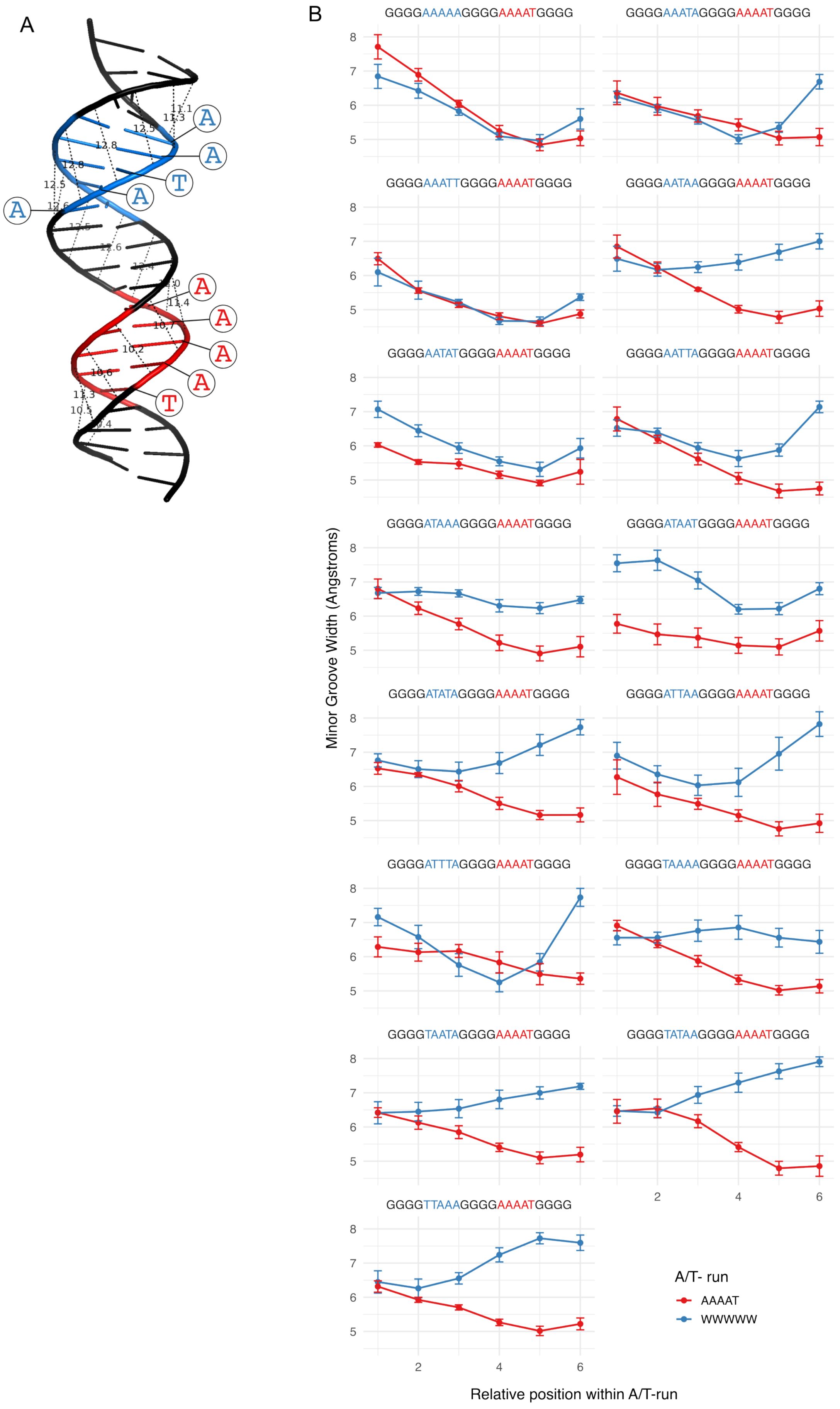
A
B
GGGGAAAAAGGGGAAAATGGGG
GGGGAAATAGGGGAAAATGGGG
GGGGAAATTGGGGAAAATGGGG
GGGGAATAAGGGGAAAATGGGG
GGGGAATATGGGGAAAATGGGG
GGGGAATTAGGGGAAAATGGGG
GGGGATAAAGGGGAAAATGGGG
GGGGATAATGGGGAAAATGGGG
GGGGATATAGGGGAAAATGGGG
GGGGATTAAGGGGAAAATGGGG
GGGGATTTAGGGGAAAATGGGG
GGGGTAAAAGGGGAAAATGGGG
GGGGTAATAGGGGAAAATGGGG
GGGGTATAAGGGGAAAATGGGG
GGGGTTAAAGGGGAAAATGGGG
Minor Groove Width (Angstroms)
Relative position within A/T-run
A/T- run
AAAAT
WWWWW

Fig. S3. AlphaFold3 A/T-run minor groove width measurements. (A) AlphaFold model 0 of 5' GGGGAATAAGGGGAAAATGGGG 3'. The angstrom width of its minor groove (black numbers) is measured as the distance between the center of the i phosphate group on one strand to the i+3 phosphate group on the complementary strand (black-dotted line). **(B)** Each plot shows the average minor groove width along A/T-runs for each 5'GGGGWWWWWGGGGAAAATGGGG 3' sequence (average of AlphaFold3 models 0-4). 5.8 angstroms are subtracted from all raw minor groove width measurements to account for the sum of the Van der Waals radii of the phosphate groups. Error bars indicate S.E.M. Position within the A/T-run (x-axis) indicates phosphate position, with position 0 being immediately 5' of the first A/T nucleotide and position 6 being immediately 3' of the last A/T nucleotide. Moving 5' to 3', we generally find that the minor groove gradually narrows along a contiguous stretch of A nucleotides, whereas sequences that start with T or switch between A's and T's inhibit this gradual narrowing. We find that AAAAA, AAAAT, and AAATT generate similarly narrow minor grooves among the A/T-runs we analyzed, yet the CBX2 AT-hook preferentially binds AAAAT. The preference for AAAAT over AAAAA likely reflects more optimal van der Waals packing in AAAAT. However, the basis for the preference for AAAAT over AAATT remains unclear and may require more detailed studies of AT-hook binding.

A

Satellite	Consensus sequence	Consensus length (bp)	Theoretical A_nT per 10 kb array	Literature
60-bp	ACATAGTATATGAGTGAGTTGCACTGAAAAACTTAGAATATAAGAAACGCACAGTGTAGT	60	0	Kipling et al 1995
79-bp	ACATTGTATATGAGTRAGTTGCACTGAAAAACTTAGA**ATTT**ACTGAAAAACCCGCATTAGAMG**AAAT**GCACAGTGTAGT	79	253	Kipling et al 1995

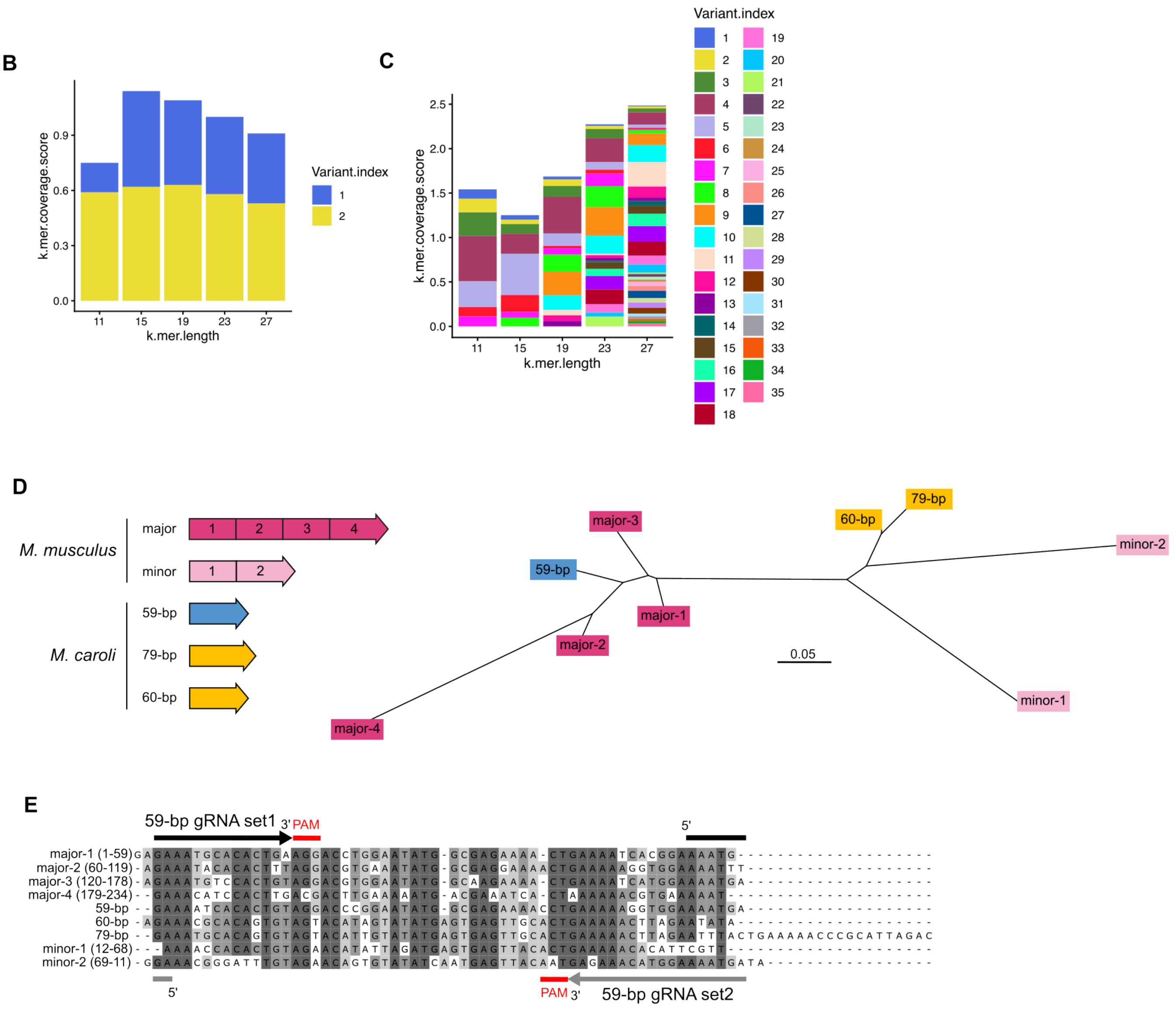

Fig. S4. The 59-bp *M. caroli* satellite is diverse and similar to major satellite. (A) Consensus sequences of the two previously identified *M. caroli* satellites (*49*) and their predicted A_nT frequencies. **(B-C)** Stacked bar plots show the number of sequence variants found for each k-mer length analysis of the 60/79-bp read cluster (B) and the 59-bp read cluster (C) and their respective coverage scores (reflecting relative abundance within the cluster). See Tables S3 and S4 for variant consensus sequences. **(D)** Left: relative lengths of the *M. musculus* and *M. caroli* satellite consensus sequences. *M. musculus* major and minor satellites are higher order repeats made up four and two sub-repeats, respectively. Right: PHYLIP Neighbor Joining tree generated from the nucleotide alignment in (E). Scale bar represents 0.05 substitutions per site. **(E)** MAFFT alignment of *M. caroli* satellites and the sub-repeats of *M. musculus* major and minor

satellites. Regions where 59-bp gRNAs anneal (black arrows) and their respective PAM sites (NGG, red lines) are indicated.

A

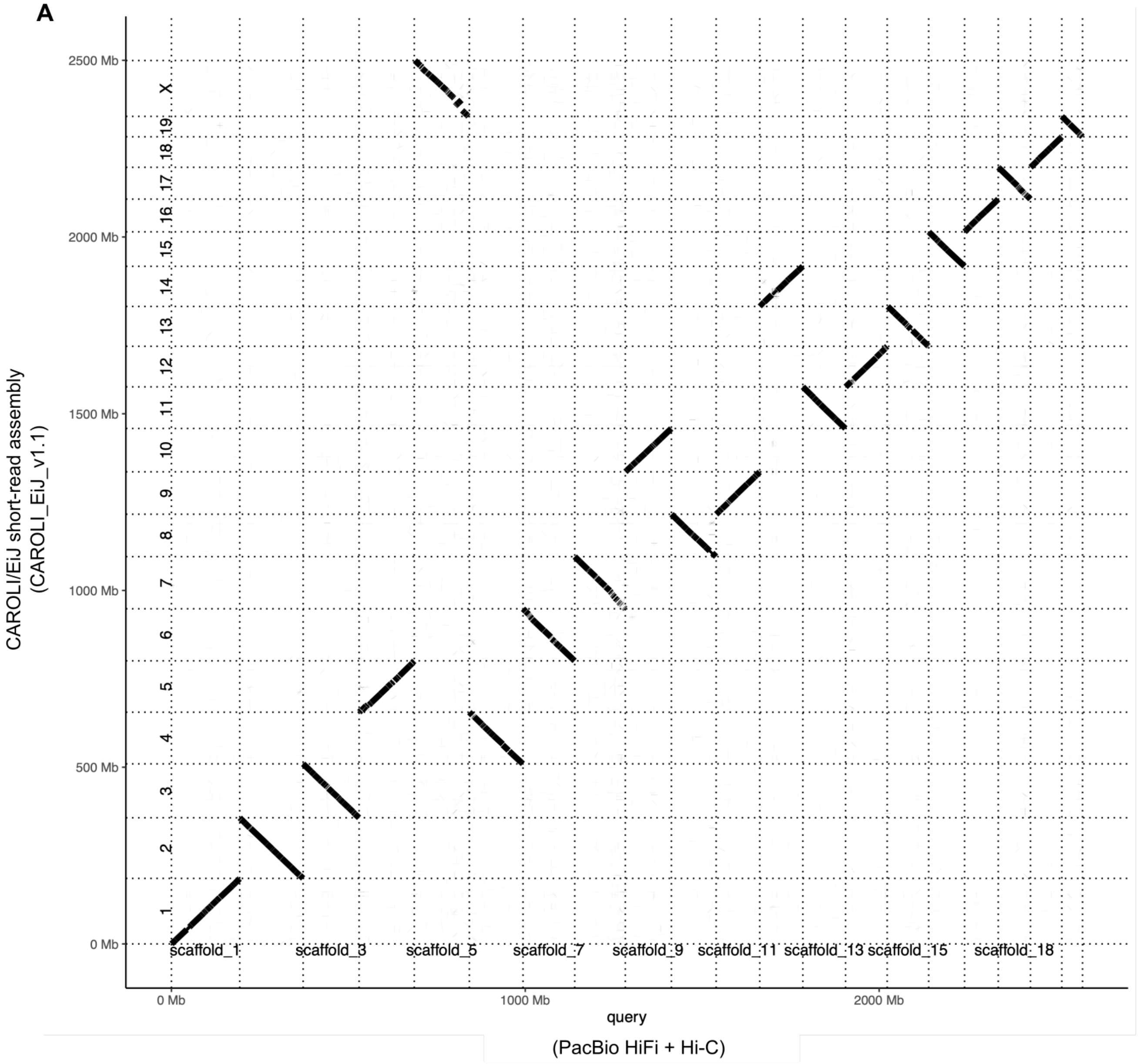

B

BUSCOs	Percent (of 9226)
Complete	99.6
Complete single copy	96.5
Complete duplicated	3.1
Fragmented	0.1
Missing	0.3

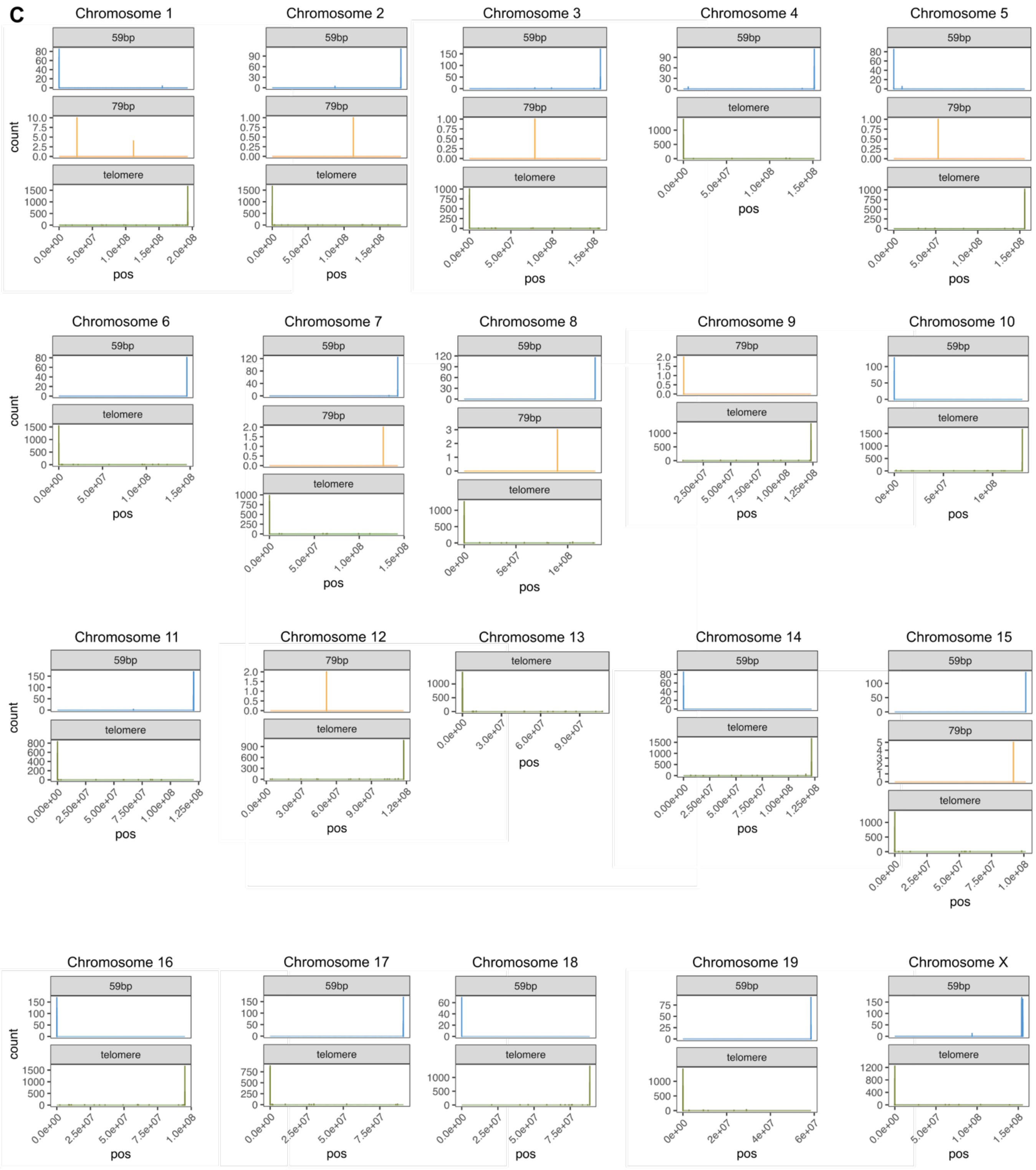
C
Chromosome 1
Chromosome 2
Chromosome 3
Chromosome 4
Chromosome 5
Chromosome 6
Chromosome 7
Chromosome 8
Chromosome 9
Chromosome 10
Chromosome 11
Chromosome 12
Chromosome 13
Chromosome 14
Chromosome 15
Chromosome 16
Chromosome 17
Chromosome 18
Chromosome 19
Chromosome X
59bp
79bp
telomere
count
pos

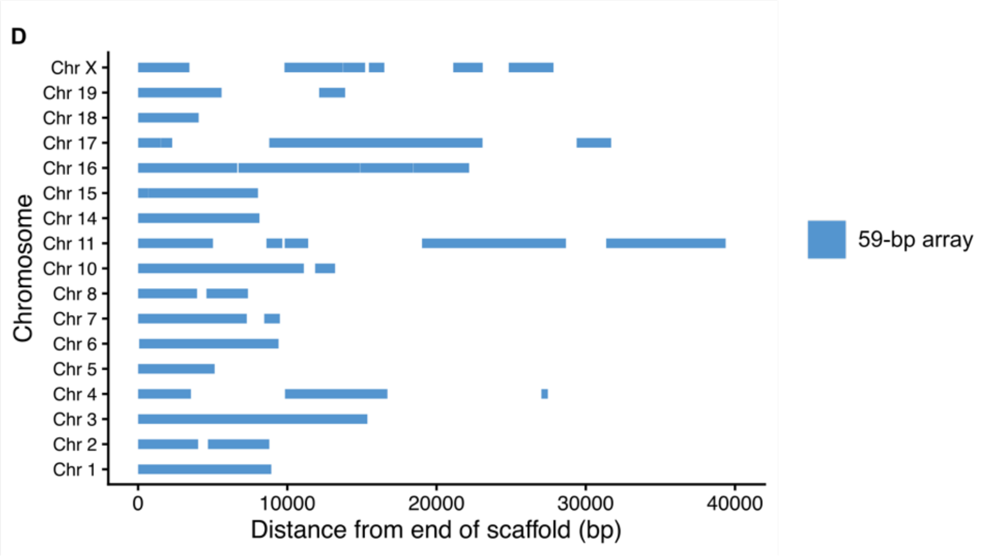

Fig. S5. Quality assessment and repeat profiling of the long-read *M. caroli* assembly. (A) Dot plot shows the alignment between our long-read *M. caroli* assembly (x-axis) and a previously published short read assembly (CAROLI_EiJ_v1.1, y-axis). Boundaries between chromosomal scaffolds in both assemblies are delineated by the dotted lines, and tick marks indicate regions of alignment between the two assemblies. Each of our chromosomal scaffolds predominantly aligns with a single chromosome from the short-read assembly. The orientation of the diagonal alignments is arbitrary, reflecting differences in chromosome orientation between the two assemblies. **(B)** BUSCO results for our long-read assembly using the mammalia_odb10 dataset. **(C)** Histograms counting the number of 59-bp, 60/79-bp and telomere (i.e. TTAGGG) repeats within 10 kb bins along all chromosomal scaffolds of our *M. caroli* long-read assembly. The x-axis label "pos" refers position in bp. **(D)** Positions and lengths of 59-bp arrays within the first 40 kb of each chromosome end in our long-read *M. caroli* assembly.

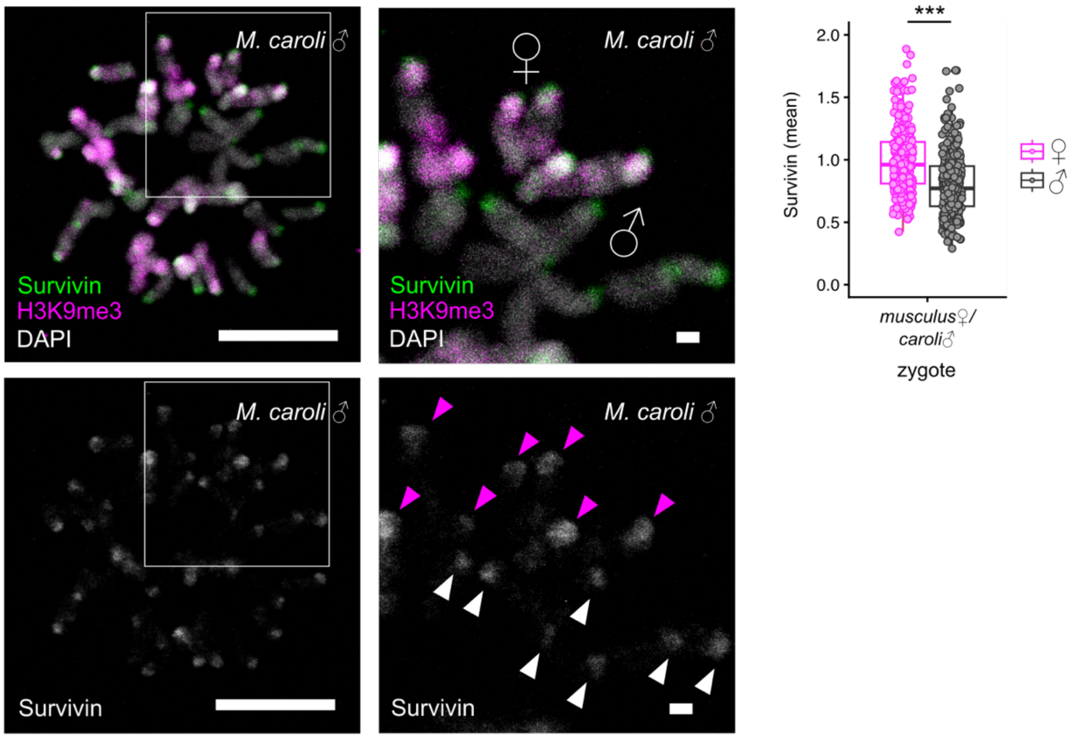

Fig. S6. Reduced CPC at paternal *M. caroli* pericentromeres. Zygotes generated with *M. caroli* sperm were arrested in mitosis with a kinesin-5 inhibitor (STLC), then fixed and stained for Survivin (green), H3K9me3 (magenta) to mark maternal *M. musculus* chromosomes, and DAPI (gray). Magenta and white arrowheads in insets point to female and male pericentric Survivin staining, respectively. Graph plots the mean Survivin intensity of maternal and paternal pericentromeres. Each point represents a single pericentromere (n=281 maternal and n=272 paternal, *N*=2) and boxes represent interquartile ranges. *P*-values were calculated by a Kruskal-Wallis test (*** $P < 0.001$). Images are max intensity z-projections; scale bars 10 µm or 1 µm (insets).

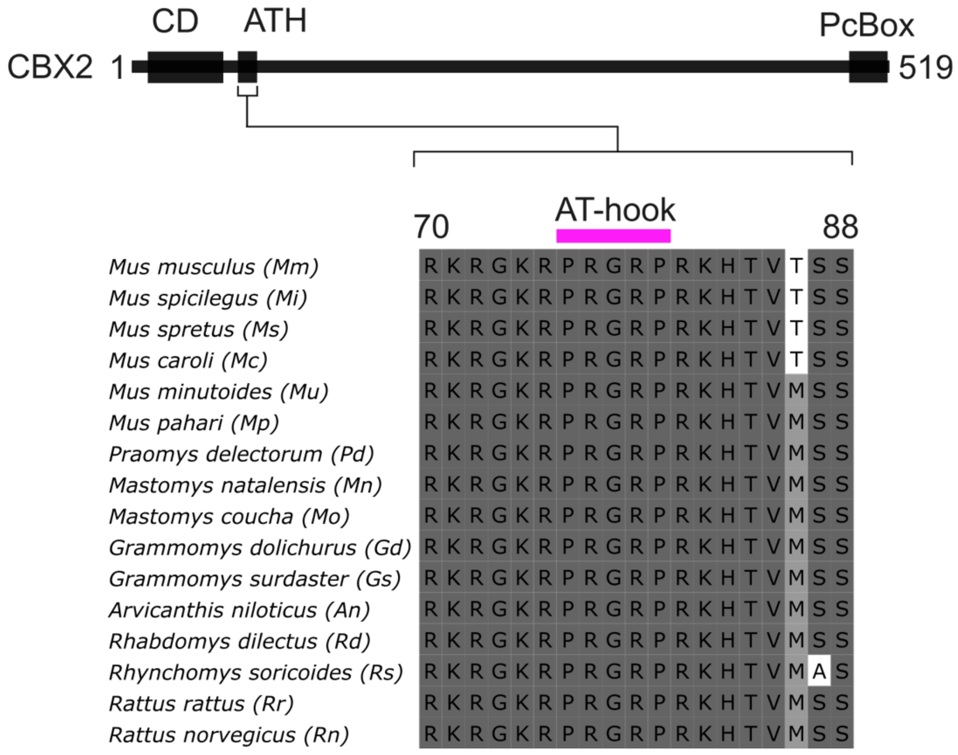

Fig. S7. Conservation of the CBX2 AT-hook across Muridae. (Top) A schematic of CBX2 protein with the position of its chromodomain (CD), AT-hook (ATH), and Polycomb Box (PcBox, PRC1 complex interaction domain) indicated by labelled black boxes. (Bottom) Amino acid alignment of the core AT-hook motif (pink bar) and flanking amino acids across 16 Muridae species (amino acid positions 70-88 in CBX2^{musculus}). Flanking amino acids make electrostatic contacts with the phosphates on the surface of the minor groove in a sequence-independent manner (*29*). Because this interaction does not depend on specific bases, the difference between *M. musculus* and *M. pahari* at position 86 is unlikely to alter CBX2 A/T-run preference.

Table S1. (Separate file) Summary of *in silico* competitive binding assays. Each row represents one of the five models ("Model output", 0-4) outputted by AlphaFold3 for a single competitive binding assay. For each model, the input DNA and CBX2 AT-hook peptide sequence are listed. The column "Competing A/T runs" lists the two A/T runs in the input DNA sequence, and the column "AT-hook bound A/T run" lists which of these two A/T runs bound the AT-hook. Confidence scores for each model are also listed (ipTM and pTM). The final column lists the four to five nucleotide sequences that the AT-hook spans for AlphaFold models containing AAAAAT. n.d. = not determined, (A?) or (T?) = uncertain whether the A/T hook spans this nucleotide.

Table S2. (Separate file) Quantification of A_nT frequency across satellite arrays and chromosome arms in *Mus* species. A table summarizing the abundance of A_nT sequences (A_3T-A_7T) in distinct genomic regions of *M. musculus*, *M. pahari* and *M. caroli*. For each region analyzed (i.e. major, pi, and 59-bp satellite arrays as well as chromosome arms) the number of A_nT, the length of the genomic region analyzed, the A_nT frequency and average number of A_nT per 10 kb are reported. Analyzed sequences are derived from whole-chromosome scaffolds for *M. caroli*, chromosome 19 for *M. musculus* (Fig 3) and h2tg000157l for *M. pahari* (Fig 3).

Table S3. (Separate file) 60/79-bp satellite k-mer report. A table summarizing the top sequence variants identified for each k-mer analysis (11-, 15-, 19-, 23- and 27-mer) in read cluster 1. The k-mer coverage score (reflecting relative abundance within the cluster), and the resulting consensus sequence generated by that k-mer are listed for each variant.

Table S4. (Separate file) 59-bp satellite k-mer report. A table summarizing the top sequence variants identified for each k-mer analysis (11-, 15-, 19-, 23- and 27-mer) in read cluster 2. The k-mer coverage score (reflecting relative abundance within the cluster), and the resulting consensus sequence generated by that k-mer are listed for each variant.

Movie S1. (Separate file). Live imaging of *M. musculus* zygotic mitosis. Live-imaging of a *M. musculus*♀/*M. musculus*♂ zygote expressing H2B:mCherry (cyan) with its microtubules labelled with SiR-tubulin (magenta) progressing through the first mitosis. Larger pronucleus contains the paternal genome whereas the smaller pronucleus contains the maternal genome. Same embryo as in Fig. 6A.

Movie S2. (Separate file). 3D reconstruction of *M. musculus* zygotic mitosis. 3D reconstruction of the zygote in Movie S1 progressing through the first mitosis. Paternal chromosomes are in blue and maternal chromosomes are in magenta.
